# Experimental considerations of acute heat stress assays to quantify coral thermal tolerance

**DOI:** 10.1038/s41598-022-20138-2

**Published:** 2022-10-07

**Authors:** J. J. V. Nielsen, G. Matthews, K. R. Frith, H. B. Harrison, M. R. Marzonie, K. L. Slaughter, D. J. Suggett, L. K. Bay

**Affiliations:** 1 College of Public Health, Medicine, and Veterinary Sciences, James Cook University, Townsville, QLD 4811 Australia; 2 Australian Institute of Marine Science, PMB #3, Townsville, MC, QLD 4810 Australia; 3 AIMS@JCU, Australian Institute of Marine Science, James Cook University, Townsville, QLD 4811 Australia; 4 Australian Research Council Centre of Excellence for Coral Reef Studies, James Cook University, Townsville, QLD 4811 Australia; 5 College of Science and Engineering, James Cook University, Townsville, QLD 4811 Australia; 6 Climate Change Cluster, Faculty of Science, University of Technology Sydney, Ultimo, NSW Australia; 7 Wellcome Centre for Human Genetics, University of Oxford, Old Road Campus, Roosevelt Drive, Oxford, OX3 7BN UK; 8 Centre for Resilience in Environment, Water and Waste, Geography, College of Life and Environmental Sciences, University of Exeter, Amory Building, Exeter, EX4 4RJ Devon UK

**Keywords:** High-throughput screening, Ecophysiology, Marine biology

## Abstract

Understanding the distribution and abundance of heat tolerant corals across seascapes is imperative for predicting responses to climate change and to support novel management actions. Thermal tolerance is variable in corals and intrinsic and extrinsic drivers of tolerance are not well understood. Traditional experimental evaluations of coral heat and bleaching tolerance typically involve ramp-and-hold experiments run across days to weeks within aquarium facilities with limits to colony replication. Field-based acute heat stress assays have emerged as an alternative experimental approach to rapidly quantify heat tolerance in many samples yet the role of key methodological considerations on the stress response measured remains unresolved. Here, we quantify the effects of coral fragment size, sampling time point, and physiological measures on the acute heat stress response in adult corals. The effect of fragment size differed between species (*Acropora tenuis* and *Pocillopora damicornis*). Most physiological parameters measured here declined over time (tissue colour, chlorophyll-*a* and protein content) from the onset of heating, with the exception of maximum photosynthetic efficiency (*F*_*v*_/*F*_*m*_) which was surprisingly stable over this time scale. Based on our experiments, we identified photosynthetic efficiency, tissue colour change, and host-specific assays such as catalase activity as key physiological measures for rapid quantification of thermal tolerance. We recommend that future applications of acute heat stress assays include larger fragments (> 9 cm^2^) where possible and sample between 10 and 24 h after the end of heat stress. A validated high-throughput experimental approach combined with cost-effective genomic and physiological measurements underpins the development of markers and maps of heat tolerance across seascapes and ocean warming scenarios.

## Introduction

Coral reefs are under increasing threat from climate change with strong and direct impacts from the interaction of chronic ocean warming^1^ and the increasing frequency of acute heat waves driving episodes of mass coral bleaching^2,3^. The process of bleaching is a well-described physiological response to the interaction of temperature and light, resulting in nutritional^4^, and oxidative stress in the coral holobiont (reviewed in^5^). It is recognised as the loss of coral colour due to expulsion of symbiotic algae and/or photosynthetic pigments^6–8^. When environmental stressors persist and/or events are extreme, bleaching may be followed by coral mortality^9,10^. Therefore, the ability of populations and species to cope with increasing temperature extremes is likely to define the structure and function of coral reefs into the future. Until recently, high throughput approaches capable of measuring and comparing heat tolerance within and between populations had only been applied to coral larvae^11,12^ and not to adult colonies^13^.

Acute thermal stress experiments provide a tool to identify and predict tolerance to stress using large sample sizes across environmental gradients^14^. In the marine environment, such experiments have been used to investigate heat stress thresholds in metabolic^15^, molecular^16,17^, and/or behavioural^18–20^ traits across a variety of marine vertebrates and invertebrates. These various approaches have identified heat-tolerant corals after exposure to acute thermal stress^21–26^. For example, Rose et al., (2018) showed that nursery corals which survived a natural bleaching event (American Samoa) all originated from top-performing colonies under acute heat stress assays. Further work in the Red Sea has demonstrated that physiological responses (including photosynthetic efficiency) from such acute assays could be differentiated among four species^14^ and were consistent with those from more traditional, longer-term heating experiments^22,26^ Consequently, acute heat stress assays are highly applicable to quantify how corals respond to different temperature treatments across broad spatial and temporal scales in the field^21,27^. However, specific experimental considerations have not been resolved for these assays.

As acute heat stress assays increase the extent of sampling possible, the processing times of ever more extensive coral phenotypic data creates an increasing bottleneck^28–30^. Existing physiological metrics of bleaching sensitivity, such as quantification of pigment (chlorophyll-*a*), protein, and antioxidative enzyme activity assays (e.g. catalase^31^) are invasive and labour-intensive to obtain. High-throughput assessment often relies on real time, non-invasive, and active fluorescence-based measures of the photo-physiological performance of coral endosymbionts—notably the maximum photochemical yield of photosystem II (PSII), *F*_*v*_/*F*_*m*_ (dimensionless^32,33^)—as a first order proxy for other physiological metrics. *F*_*v*_/*F*_*m*_ is long evidenced in quantifying declining endosymbiont function as corals bleach under heat stress^33,34^, and correlates to other heat-response characteristics such as declining chlorophyll-*a* content^35^, protein content^36^, and changes to the microbial community composition^37^. Other studies have employed image-based measures of colour to rapidly assay bleaching^38^; for example, Nielsen et al.^39^ showed a strong relationship between tissue colour and chlorophyll-*a* content. Thus, coupling readily quantifiable, cost-effective parameters and their relationship to thermal tolerance with acute heat stress assays allows faster quantification of the coral bleaching response and provides a platform for developing a deeper insight into patterns of thermal tolerance across time and space.

To ensure that growing acute heat stress data sets^14,21,26^ are comparable among studies to support robust reconciliation through cross-study meta-analyses, a consistent set of guidelines will be required. A common standardised framework is required to resolve drivers of bleaching susceptibility between species and regions spanning different geographical (habitat, reef, region) and biological (colony, population, species) scales^40^. Basic operational factors that can potentially influence measures of thermal tolerance remain largely untested^41,42^. The size of the sampled fragment has been shown to affect thermal tolerance and bleaching resistance in some corals^43,44^. Similarly, it is unknown whether physiological changes occur linearly or non-linearly over time, and by extension, whether studies measuring at different time points can be compared^45,46^.

We examined how the understanding of heat tolerance based on acute assays is affected by fundamental methodological considerations. We firstly investigate the effect of experimental fragment size using two common coral species of varying thermal sensitivities, *Acropora tenuis* and *Pocillopora damicornis*. Since published studies of acute thermal tolerance have sampled at slightly different time-points, we then examined the effect of sampling time on the resulting acute heat stress phenotypes of *A. tenuis* over 48 h. Due to the high-throughput potential of these acute heat stress assays, we provide a cost analysis of the physiological metrics included here and finally we investigate how rapid, non-invasive measures of coral thermal tolerance (*F*_*v*_/*F*_*m*_ and colour change) compare to more time-consuming and labour-intensive measures using evidence from multiple physiological traits. We discuss experimental considerations and cost effectiveness of physiological measurements for future applications of high throughput acute heat stress assays to measure thermal tolerance of corals and identify rapidly quantifiable descriptors of physiological responses to heat stress. This study benefits the development of cost-effective and rapid descriptors of (heat) stress tolerance amongst coral populations for targeted protection or propagation.

## Results

### Experiment 1: Effect of fragment size

**Figure 1:**
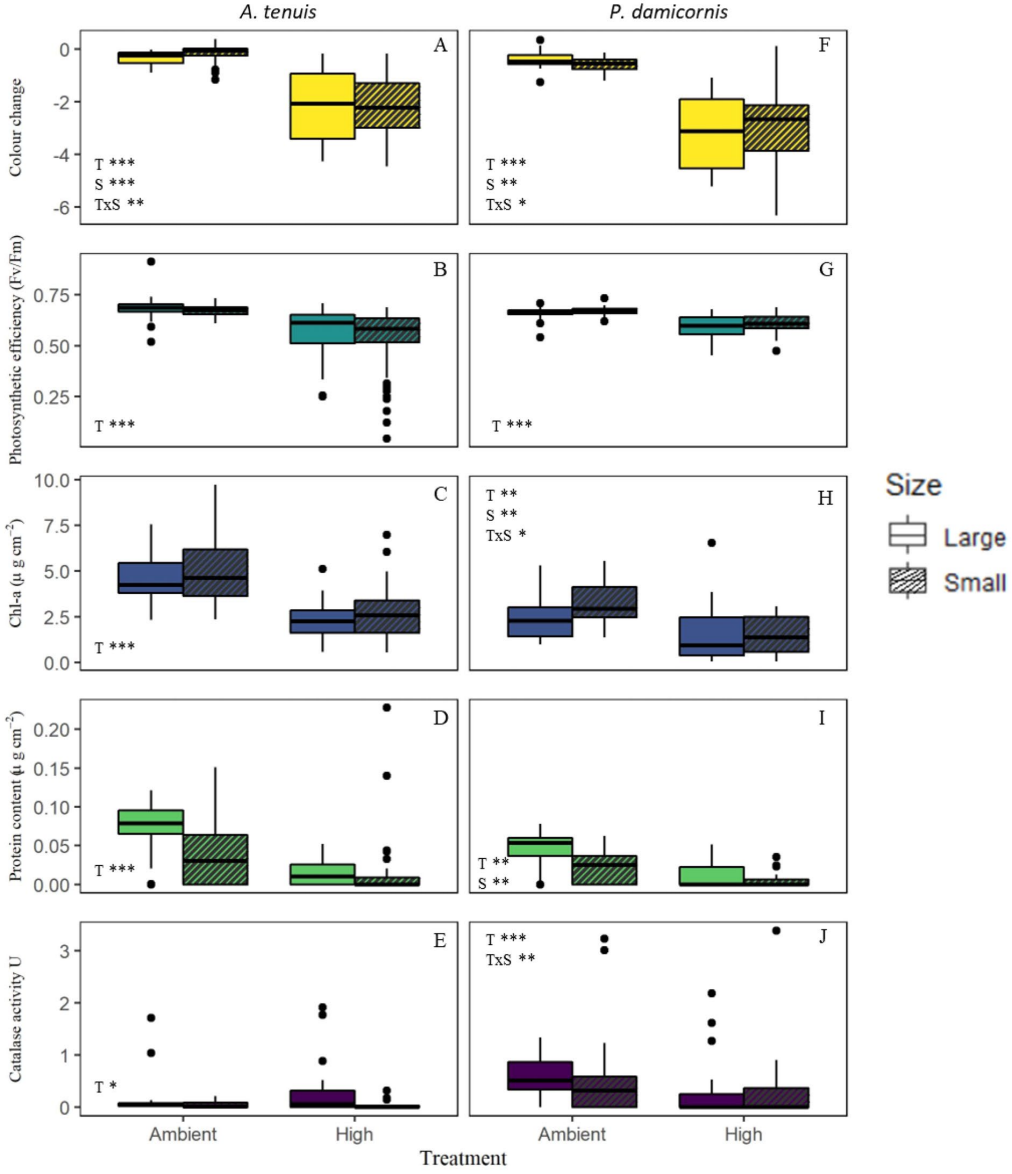
Physiological responses of large (full) and small (hatched) coral fragments to temperature treatment in *A. tenuis* (left panels) and *P. damicornis* (right panels). Bold line inside boxes shows the median, boxes indicate the interquartile range and dots represent data outliers. Significant effects are indicated for treatment (T), size (S) and their interaction (T × S) by asterisk where *p < 0.05, **p < 0.005, ***p < 0.0005.

Effect of fragment size differed between species and physiological metrics investigated. Collectively, fragment size affected nearly all examined physiological measures in *P. damicornis* except photosynthetic efficiency while an effect of fragment size was largely absent in *A. tenuis* samples. Treatments at high temperatures resulted in significant declines across all measures relative to treatments at ambient temperatures.Tissue colour change was affected by the interaction of treatment and fragment size in both species (*A. tenuis*, df = 106, z = − 3.26, *p* = 0.001; *P. damicornis*, df = 106, z = 2.50, *p* = 0.023). In *A. tenuis*, large fragments (− 0.34 ± 0.05 colour units) exhibited nearly twice the colour loss of small fragments (− 0.19 ± 0.07 colour unit, df = 106, t = − 4.231, *p* < 0.0001, Fig. 1A) while in *P. damicornis*, large fragments (− 0.40 ± 0.06 colour unit) exhibited less colour loss relative to the small fragments (− 0.91 ± 0.06 colour unit, Fig. 1F, df = 106, t = 5.745, *p* < 0.0001). See statistical outputs in Supplementary materials S3.1 and S3.2.

Chlorophyll-*a* content and catalase activity (U) in *P. damicornis* were both affected by the interaction of treatment and fragment size (Chl-α; df = 96, z = − 1.975, *p* = 0.048, Fig. 1H; catalase; df = 86, z = 2.82, *p* = 0.005, Fig. 1J, respectively). Catalase activity (U) varied within heat-treated corals between fragment sizes (df = 86, t = − 2.146, *p* = 0.035; large = 0.286 ± 0.1 U; small = 0.03 ± 0.01) but not for ambient corals (df = 86, t = 1.851, *p* = 0.068). In *A. tenuis*, chlorophyll-*a* content and catalase activity were only affected by temperature (chlorophyll-*a* df = 104, z = − 6.24, *p* < 0.001, Fig. 1C; catalase activity df = 93, z = 2.38, *p* = 0.017, Fig. 1E) but not fragment size, or their interaction.

Photosynthetic efficiency was only affected by treatment in both species, but not fragment size or their interaction (Fig. 1B,G). Finally, *P. damicornis* protein content was affected by both treatment and fragment size, but not by their interaction (Treatment; df = 102, z = − 3.173, *p* = 0.002; Size df = 102, z = − 2.761, *p* = 0.0058, Fig. 1I). In *A. tenuis*, protein content was only impacted by treatments (df = 91, z = − 5.112, *p* < 0.0001, Fig. 1D).

### Experiment 2: Time effect

**Figure 2:**
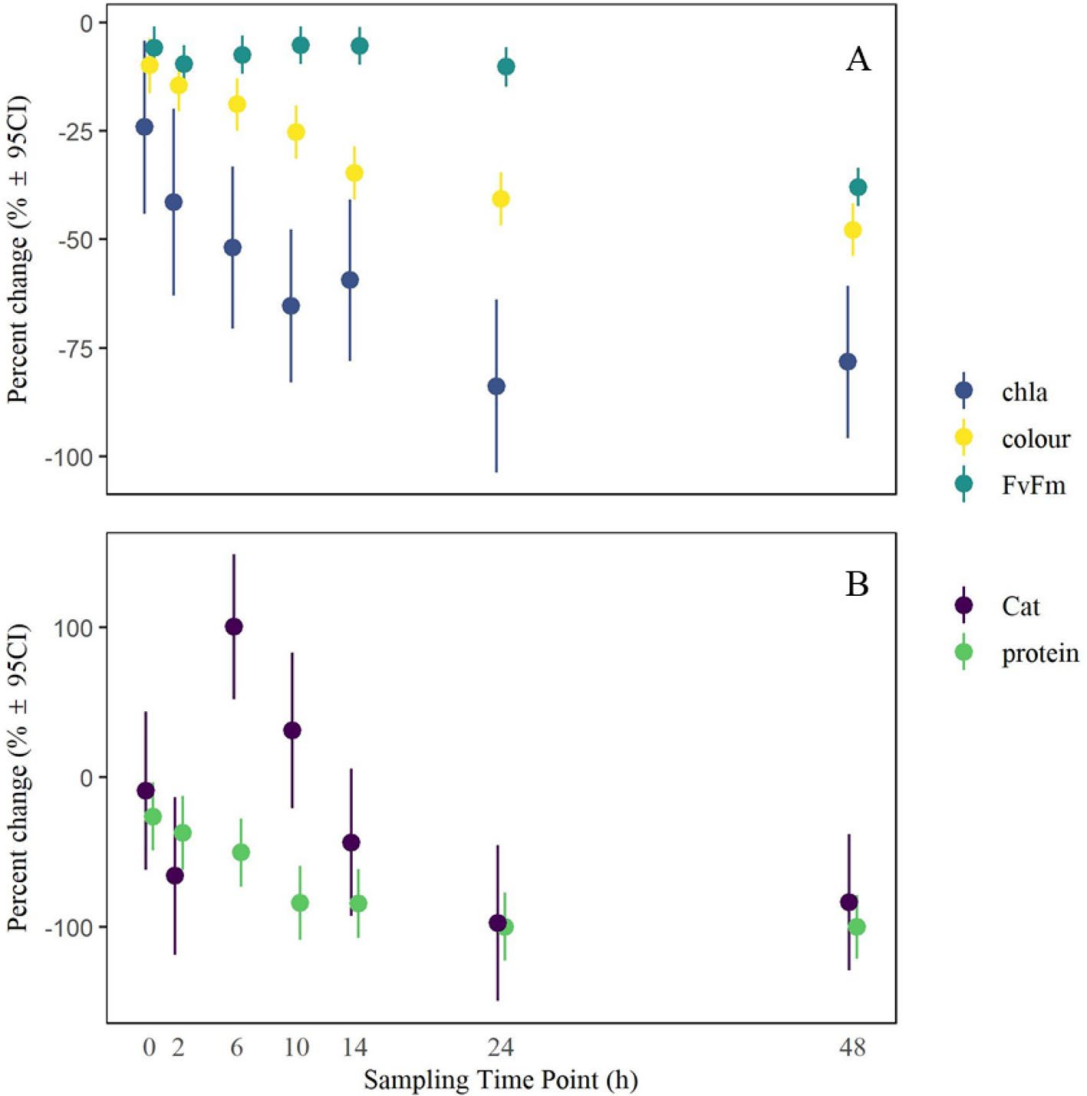
Percent change in physiological metrics over time in heat-treated relative to ambient corals. Fragments from nine colonies were sampled through time at 0–48 h (T_0_–T_6_) after the end of heat stress from both an ambient (29.6 °C) and heated treatment (34.6 °C). (**A**) Fluorometric and colour assays. (**B**) Biochemical assays. Points represent the estimated marginal means of physiological metrics at each sampling time point (T_1_–T_6_). Error bars indicate the upper and lower 95% confidence intervals.

All physiological metrics except catalase experienced a significant initial decline immediately following heat stress. Most metrics then continued to decline through time before reaching a steady-state between 10 and 24 h after heat stress. However, photosynthetic efficiency was stable until the final sampling point at 48 h (Fig. 2). Coral tissue colour recorded an initial decrease immediately following the exposure to heat stress (0 h, T_0,_ − 9.98%, z = − 3.18, *p* = 0.0015), and remained stable until 6 h and then declined steadily until 24 h (T_5_; post hoc T_2_–T_5_ t = 7.57 *p* < 0.001) before stabilising again and remaining unchanged until 48 h (T_6_, Fig. 2A, T_5_–T_6_ t = 2.51, *p* = 0.18). Similarly, chlorophyll-*a* content declined at 0 h (T_0_, − 24.18%, z = − 2.44, *p* = 0.015) before stabilising at 10 h (T_3_, Fig. 2A). In contrast, photosynthetic efficiency (*F*_*v*_/*F*_*m*_) declined by 5.8% immediately following the experiment (z = − 2.4, *p* = 0.016) and remained stable until the 24-h sampling point (T_5_, Fig. 2A) before declining again after 48-h (T_6_, Fig. 2A).

Antioxidative catalase activity did not change initially following exposure to heat stress but recorded a significant increase 6 h after heat stress (T_0_–T_2_, t = − 3.24, *p* = 0.037). Catalase activity then decreased towards the end of the experiment and was nearly absent by 24 h (T_5_, − 88.38%, Fig. 2B). Finally, protein content recorded an initial decrease immediately following heat exposure (26.44%, z = − 2.35, *p* = 0.019) and then declined in the first 10 h (T_0_–T_3_ t = 3.48, *p* = 0.02) before stabilising (− 58.1%, Fig. 2B). All statistical outcomes from post-hoc comparisons are presented in Supplementary material S4.2.

### Experiment 3: Physiological measures comparisons

**Figure 3:**
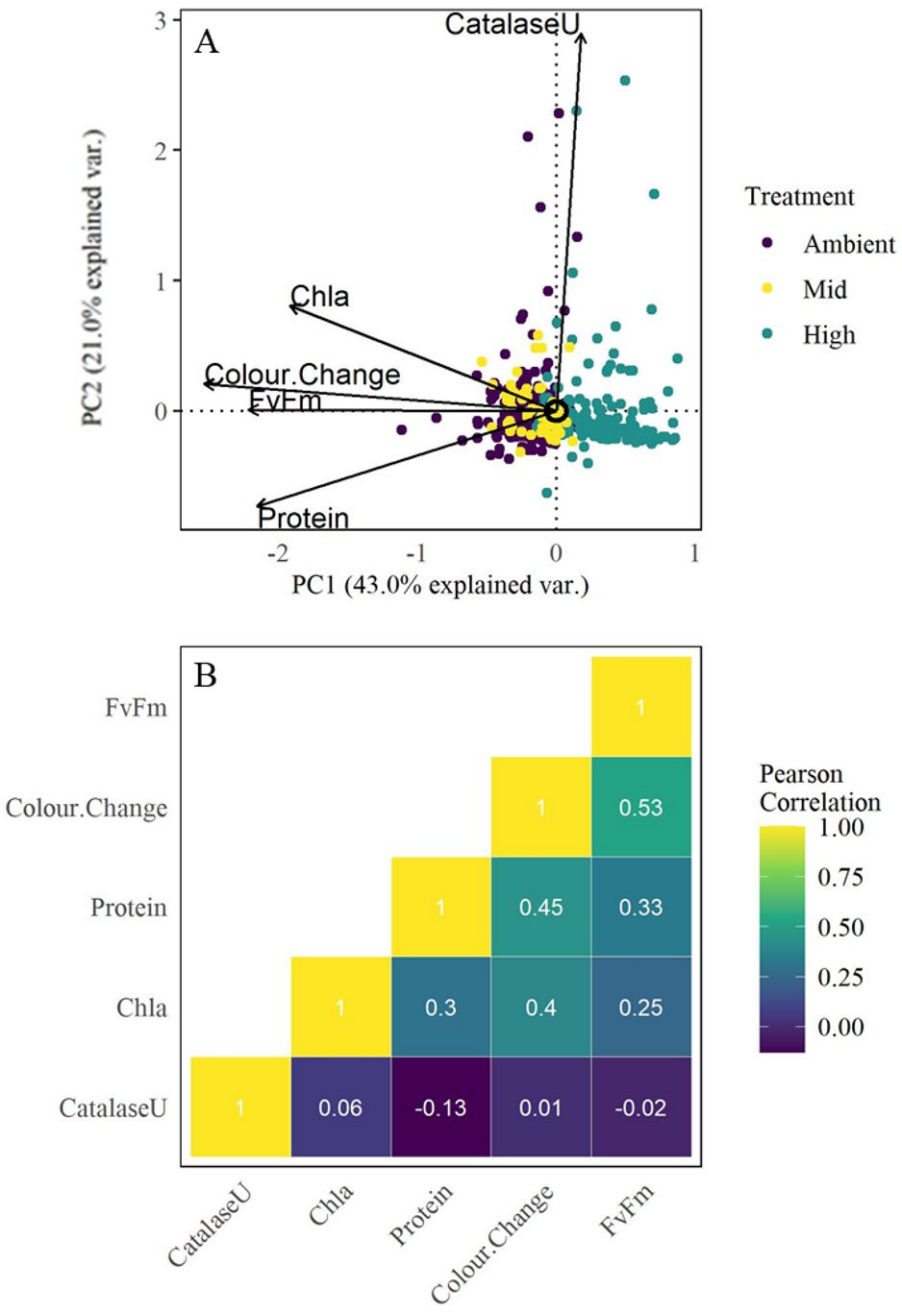
Relationships between multiple physiological responses to heat stress in *A. tenuis*. (**A**) Principal Component Analysis (PCA) of five physiological traits in response to acute heat stress in *A.tenuis* (n reefs = 7, n samples = 423) and (**B**) correlation heatmap between all traits. Yellow diagonal are self-comparisons.

To investigate how rapid, non-invasive measures of coral thermal tolerance (*F*_*v*_/*F*_*m*_ and colour change) compared to more time-consuming and labour-intensive measures, we performed a Principal Component Analysis. The PCA identified response patterns of multiple physiological measures to acute thermal exposure in *A. tenuis* (Fig. 3). Four of the five physiological response measurements (colour change, protein and chlorophyll content, and *F*_*v*_/*F*_*m*_) were correlated to and accounted for variation along PC1 (43% variation explained), while only catalase activity separated data along PC2 (21% variation explained). All physiological metrics analysed were significant in driving the separation among samples (Fig. 3A).

To identify which metrics were driving data variability we examined correlations among the physiological metrics (Fig. 3B). Both tissue colour change and maximum photosynthetic efficiency (*F*_*v*_/*F*_*m*_) showed a significantly positive correlation to the three laboratory-derived metrics (catalase, protein, and chlorophyll-*α* content). Both tissue colour change and *F*_*v*_/*F*_*m*_ were most strongly correlated with host protein content. As such, both non-invasive physiological measures of tolerance describe overall patterns observed.

### Cost–benefit analysis

Of all physiological measurements utilised here, the more rapid field-based measures of maximum photosynthetic efficiency (*F*_*v*_/*F*_*m*_) and tissue colour change carried the lowest associated costs (including labour) and were also the most time-efficient (Tables 1, 2). Based on 100 samples, we estimated a time of ~ 45 min to gather and a further 45 min to analyse maximum photosynthetic efficiency data and ~ 100 min to gather and analyse tissue colour changes. In comparison, > 28 h was required to quantify protein content in the laboratory with catalase and chlorophyll assays each requiring approximately 25 h to complete.

**Table 1:** Cost of consumables and time requirements for each assay to process 100 samples.

Assay	Consumable cost 100 samples ($AUD)	Time requirement 100 samples (min)	Time cost @$33 p/h	Total
Photosynthesis efficiency (PAM)	NA	90	$49.5	$49.5
Tissue colour change	NA	102	$56.1	$56.1
Tissue blasting	$156	1210	$665.5	$821.5
Chlorophyll	$27	305	$167.75	$194.75
Protein	$49	463	$254.65	$303.65
Catalase	$85	480	$264	$349
Surface area	$4.2	585	$321.75	$325.95
Total for 100 samples	$321.2	54 h	$1782	
Grand total				$2103.2

**Table 2:**
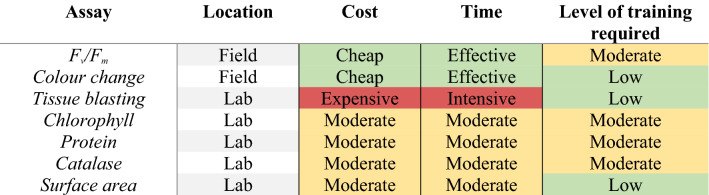
Costs and benefits of measures of coral thermal tolerance.

Benefit classification used based on 100 samples; financial; Cheap < $200, Moderate $200–$600, Expensive > $600. Time; Effective < 5 h, Moderate 5–10 h, Intensive > 10 h per 100 samples. Level of training required; low = little to no instruction required, easy to do from protocol, no specialised laboratory skills required; Moderate = some basic laboratory skills required, operator generally supervised a couple of times then works from protocol, special instruction in equipment use. Hourly rate used for time cost is $33 AUD per hour. See Supplementary Material S6.1 for an overview and price-guideline for the equipment required for each of these assays. Cell colours reflect coding for high (red; expensive, intensive), medium (yellow; moderate) and low (green; cheap, effective, low) across categories.

## Discussion

Variation in coral thermal tolerance both within-^47,48^ and between-^49^ reef systems is likely key to their continued survival under further ocean warming^23,50,51^. To date, aquarium-based ramp and hold experiments have been widely applied to investigate variation in thermal tolerance but are limited logistically in terms of how many samples can be included and the sampling areas they can cover. Recently, acute heat stress assays have increased the capacity to quantify heat tolerance in adult corals (e.g.^21,26^) through field-deployments with rapid experimental turnover. However, efforts to scale towards higher throughput both within studies and through comparisons among studies must be based on solid methodologies that control technical sources of variance and utilise common measures of coral thermal tolerance^13,40^.

Our study investigated the effect of fragment size and sampling timing on coral acute thermal tolerance. We presented a cost analysis of all physiological measures analysed herein to provide planning background to other users of acute thermal stress assays and finally, we showed how rapid, non-invasive measures of coral thermal tolerance (*F*_*v*_/*F*_*m*_ and tissue colour change) compared to more time-consuming and labour-intensive measures using evidence from multiple physiological traits. Together, our results highlight the need to consider fundamental experimental design criteria of these assays to ensure that results are repeatable and comparable among studies.

### Fragment size affected P. damicornis more than A. tenuis

There are currently no guidelines on appropriate fragment sizes for experimental examination of coral thermal tolerance^13^ and this metric is rarely reported^52^. The coral restoration literature has suggested that larger fragments may result in greater survival^53^ although this is not always the case^54–56^. With the advance of acute heat stress assays, fragment size could therefore be a source of technical variability. In our study, effect of fragment size differed between species; one out of five physiological responses of *A. tenuis* were significantly affected by fragment size while in *P. damicornis* four out of five measures showed significant fragment size effects. In *A. tenuis*, large fragments showed greater colour loss than the small fragments in the ambient treatment. Similarly, fragments of *Acropora palmata* have recorded differential bleaching resistance during a natural thermal stress event where small fragments recorded less bleaching than larger ones^43^. Additionally, corallite formation differs between the two species where *A. tenuis* produces an apical corallite characteristic for the *Acroporids* while *P. damicornis* does not. The presence of an apical corallite in the absence of Symbiodiniaceae-rich tissues could potentially skew the colour change metric. Whilst we did not test this factor explicitly, it would be important in the future to consider species-specific morphology when designing data-gathering protocols that span diverse taxa.

Changes in protein content and catalase enzyme activity were more pronounced in large relative to small fragments in *P. damicornis*. Protein and catalase assays from small fragments may be nearing the detection limits of the instrument (spectrophotometer), and the issue is further compounded by quantifying surface area by wax dipping as uncertainty increases when used on small fragments^57^. To avoid potential detection limits of assays and instruments, we recommend using larger fragments (~ 9 cm^2^ for *A. tenuis* and ~ 12 cm^2^ for *P. damicornis*). Ultimately, properties that require normalisation—and therefore introduce error propagation from > 1 measurement—may be less suitable to detect more subtle changes through acute stress experimentation. Interestingly, size effects primarily occurred in ambient-treated corals and were absent in heat-treated fragments, suggesting that a fragment size effect is introduced to the experiments initially, but that this effect is insignificant compared to the applied heat exposure. While this is important to consider when comparing heated to non-heated coral fragments, we demonstrate no size effect on physiological responses in either species in the heated treatment, highlighting that any initial size effects are not likely to influence thermal tolerance results obtained by this approach.

### Choosing when to sample post heat stress impacts conclusions drawn

Responses to heat stress varies with exposure duration and sampling time. Sampling variation may therefore limit how different combinations of measurements can ultimately be used to reconcile large-scale heat stress assay data sets. Therefore, sampling time is a critical component of experimental design. Here we observed that physiological responses decreased up to 24 h post heating with the notable exception of photosynthetic efficiency which was stable up to 24 h. We therefore recommend sampling between 10 and 24 h post heating. Sampling prior to 10 h post heating could fail to detect a response while sampling post 24 h may result in sampling of mortality or severe tissue necrosis, particularly at higher temperatures (see^58^). We did not sample past two days post heating (48 h) to maintain the rapidity of these acute heat stress assays. Other studies have also reported rapid changes in response to acute heat stress; for example, Dove et al.,^59^ found significantly reduced protein and chlorophyll content following a 6-h temperature exposure while Traylor-Knowles et al.,^60^ found upregulated heat shock protein expression in response to heat stress after only 2 h 30 min and evidence of protein degradation after 5 h. The decline through time observed in most traits in the present study may complicate direct comparisons of results between studies depending on the traits quantified and the sampling time point.

### Time- and cost- efficient physiological measures to capture coral thermal tolerance variability

Capitalising on the rapid throughput of acute heat stress assays requires the adoption of standardised phenotyping measures which can be quantified rapidly in the field at minimal cost. Maximum photosynthetic efficiency (*F*_*v*_/*F*_*m*_) and tissue colour change are both time- and cost-efficient to capture, making them ideal candidate measurements for rapid tests of coral thermal tolerance. While photosynthetic efficiency was the fastest measure quantified in this study, the capital outlay for a fluorometer such as the one used here (~ $50,000 AUD) may be beyond the scope of some groups. However, cheaper alternatives exist (for example AquaPen®, ~ $4050 AUD) and the costings presented here are highly conservative. While fluorometric data is fast to gather and has a low cost per sample when considering the lifespan of the instrument, it is not currently possible to calibrate fluorometric data between studies due to the lack of universal standards, multiple sensor types and diverse sampling protocols used^30,61^. This makes direct comparisons between studies challenging.

When considering capital costs and accessibility, tissue colour change is by far the most cost-effective measure captured, further reducing processing and analysis time investment through the development of automated approaches^62^. Recent technological advances also allow scaling of automated bleaching assessments with the implementation of new technologies such as hyperspectral imaging^63^, despite additional and significant capital costs. If these rapid measures (tissue colour change and *F*_*v*_/*F*_*m*_) are to be used at a large scale, it is important to keep their relationship to coral thermal tolerance in mind and carefully consider which measures best address the research question.

### Selecting physiological measures of coral thermal tolerance for acute heat stress assays

Photosynthetic efficiency and tissue colour change are higher-order traits, derived from multiple other measures. For example, changes in tissue colour can result from a loss of Symbiodiniaceae cells, loss of chlorophyll pigmentation within those cells^38^, or the loss of coral tissue itself. Photosynthetic efficiency, on the other hand, is a direct measure of viability of the symbiont partners and only an indirect indicator of thermal tolerance of the coral holobiont^8,45^. We therefore examined whether tissue colour change and photosynthetic efficiency captured differences in other physiological measures of thermal tolerance in *A. tenuis*. We found that both photosynthetic efficiency and tissue colour change showed similar responses to heat stress as chlorophyll-*a* and protein content but not catalase activity. Similarly, acute heat stress studies of *Stylophora pistillata*^22^ found that changes in *F*_*v*_/*F*_*m*_ values correspond well to other physiological measures quantified and a high correlation between *F*_*v*_/*F*_*m*_ and tissue colour change was reported in *Siderastrea siderea*^64^. Coral host catalase activity showed an opposite trend to all other measures as catalase was correlated to PC2 rather than PC1 (Fig. 3A). The opposing trend displayed by the catalase vector in the PCA is expected as catalase generally increases during heat stress^31,65^, while all other measures quantified here are expected to decrease. As a mechanistic measure of heat tolerance, catalase activity or other antioxidative enzymes provide valuable insight into the host responses to acute heat stress^31^ but are impractical and time-consuming for ‘routine’ use of high throughput assays. Due to the scalability of acute heat stress assays, it is also possible to utilise these experiments for higher throughput mechanistic studies including metabolomics, proteomics and gene expression analyses^58,66^.

Finally, when selecting which physiology traits to measure for acute heat stress assays, it is important to consider the data variability that appears inherent with this experimental approach. We document large standard errors in all responses despite sampling > 110 colonies. However, this is also the case for other acute heat stress assays^26,27^. As such, alternate indicators of thermal tolerance may have different capacities to resolve subtle differences in temperature thresholds^14^.

Acute stress experiments have resolved thermal tolerances of many organisms including fish^67,68^, intertidal invertebrates^69^, extremophiles^70^, and coral; both in adult life stages^26^ and larvae^12,71^. Here, we assess aspects of experimental design for acute heat stress assays and their applicability to coral studies. We suggest that sampling occurs more than 10 h after the end of heat stress but before the 24 h mark. We conclude that by adopting standardised approaches, these experiments have the capacity to address the yet unresolved mechanisms of thermal tolerance and provide a means to obtain information spanning emergent physiological responses and thermal thresholds to underlying transcriptional regulation^72^ and form the basis in moving towards a systems biology approach^73^. If large datasets are collected across spatial and temporal scales, insights such as environmental and genomic drivers of tolerance and thermal adaptation could be identified. Scaling efforts to quantify thermal tolerance is becoming increasingly important due to the continued threat to coral reefs globally from climate change.

## Materials and methods

### Study region, species, and collection

**Figure 4:**
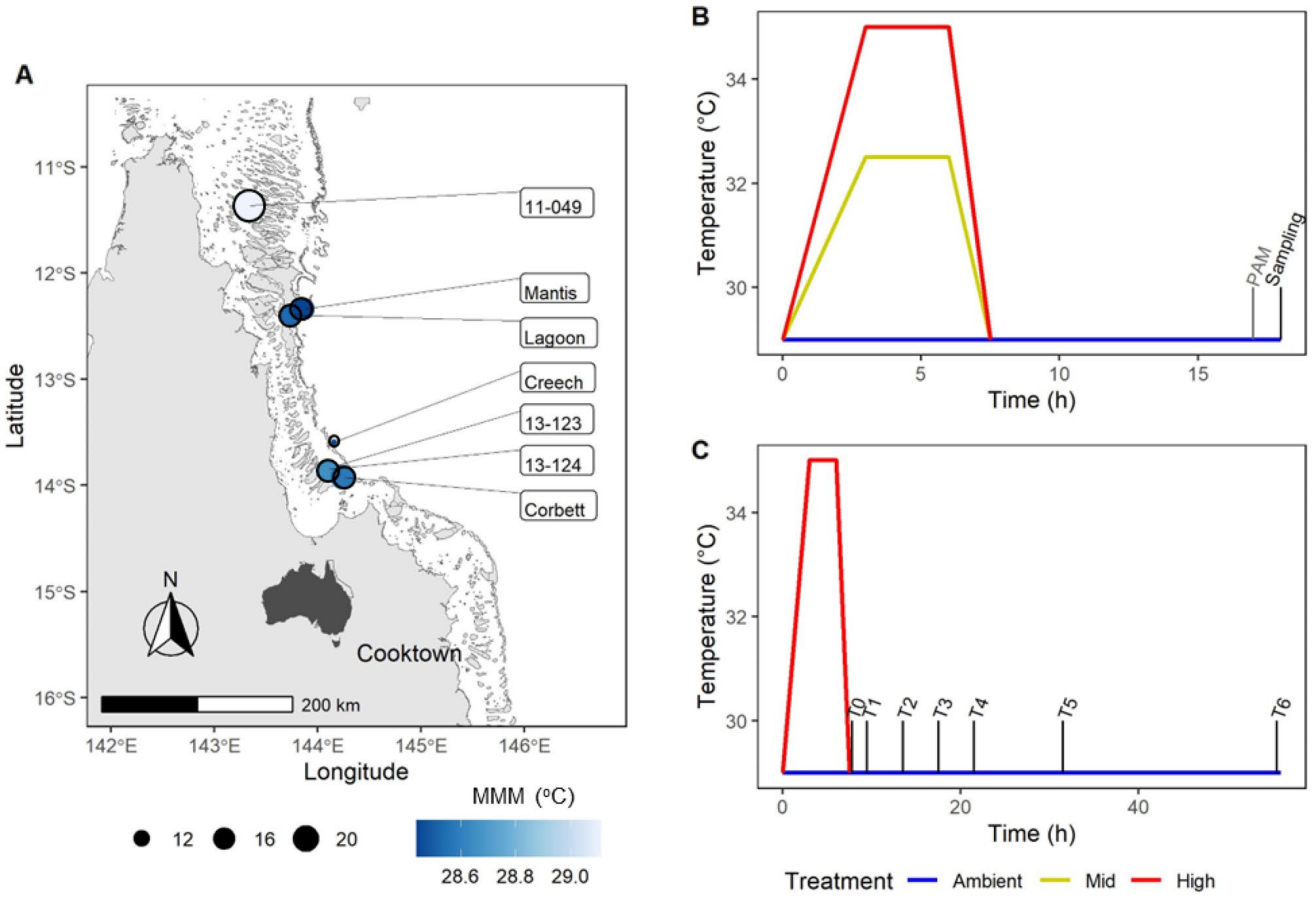
Collection and experimental designs used to examine the influence of fragment size on two species (*A. tenuis* and *P. damicornis*) and time of sampling on multiple physiological measures. (**A**) Map of sampling locations, size of dot indicates number of colonies sampled per site and colour shows Max Monthly Mean (MMM) temperature of each site. Map generated in R version 4.1.3. (**B**) Temperature profiles used to test for size effects in *A. tenuis* and *P. damicornis* (ambient and high treatments only, experiment 1) and to investigate multiple physiological measures across five reefs in *A. tenuis* (all three treatments, experiment 3). (**C**) Temperature profile and sampling time points for assessing changes in physiological metrics through time (experiment 2).

Acute heat stress assays on *Acropora tenuis* and *Pocillopora damicornis* were conducted in the Far Northern Great Barrier Reef (FNGBR) in January 2019 (Fig. 4A). This region of the Reef is characterised by high summer temperatures and irradiance^74,75^ and experienced consecutive bleaching events in the austral summers of 2016 and 2017 (18–82% bleaching, n = 15 reefs^3^). All coral samples were collected on SCUBA (3–6 m) under Great Barrier Reef Marine Park permit G16/38488. Colony colour was assessed against the Coral Watch reference chart at the time of collection^76^. Fragments were placed in perforated zip-lock bags for no more than 2 h, and further fragmented for experiments. Fragments were placed in aquaria (60L) on the vessel deck, supplied with ambient flow-through seawater, and shaded with cloth prior to being moved into the experimental tanks. Seven separate experimental runs were conducted no later than 24 h after collection across three experiments (Table 3). The two species were selected to represent two abundant genera and for their ease of collection with hand tools. Collection, transport, and fragmentation are likely stressful for corals and our protocol did not allow for recovery time prior to experimental exposure, hence heated fragments were compared to ambient-held fragments.

**Table 3:** Coral collection and experiment details.

Purpose of experiment	Reef	Coordinates	Species collected	Colonies sampled	Treatments	MMM °C	Collection date	Experiment date
1. Fragment Size effect	13–123	144.1348°E, 13.8552°S	*A. tenuis*	9	Ambient and + 6 °C	28.6	14/01	15/01
			*P. damicornis*	9				
2. Time effect	Creech	144.1071°E, 13.6447°S	*A. tenuis*	9	Ambient and + 6 °C	28.46	15/01	16/01
3. Alternative physiological measurements	Corbett	144.2405°E, 13.9227°S	*A. tenuis*	18	Ambient, + 3 °C, + 6 °C	28.58	10–11/01	11/01
	13–124	144.0906°E, 13.8517°S	*A. tenuis*	15	Ambient, + 3 °C, + 6 °C	28.66	12–13/01	13/01
	Lagoon	142.7394°E, 12.3922°S	*A. tenuis*	15	Ambient, + 3 °C, + 6 °C	28.54	18/01	19/01
	Mantis	143.8808°E, 12.3041°S	*A. tenuis*	15	Ambient, + 3 °C, + 6 °C	28.44	20/01	21/01
	11–049	143.3262°E, 11.3637°S	*A. tenuis*	23	Ambient and + 6 °C	29.11	28/01	28/01

Collection dates are given as day of January 2019.

### Temperature treatments and experimental design

Heat treatment profiles were designed following Palumbi et al.^77^ and Voolstra et al.^26^ using a new delivery system designed by the National Sea Simulator Facility at the Australian Institute of Marine Science (AIMS, Supplementary material S1). The temperature manipulation system was run indoors onboard a research vessel and consisted of an initial ramp up over 3 h from ambient incoming seawater to the desired treatment temperature. Treatment temperature was held for 3 h, followed by ramp down to ambient within 1.5 h. Once returned to ambient temperature, corals were maintained for 11 h in the dark before data collection and sampling (Fig. 4B). The control treatment was held at ambient temperature for the duration of the experiment and ambient temperatures ranged between 29.5 and 30.9 °C across the sampling duration. Treatments used were ambient, mid (approx. + 3 °C) and high (+ 6 °C). Experimental temperatures and Max Monthly Mean (MMM) temperatures are presented in Supplementary material S2. Lighting profiles followed summer, mid-day light levels at Lizard Island in the northern GBR (450 µmol photons m^−2^ s^−1^, no ramping, 7 h:11 h light:dark, 60% blue, 20% white, 10% green, and 10% red, 10 M, Lizard Island Light From 26 Feb 2012 | AIMS metadata | aims.gov.au). We conducted three separate experiment to test the effects of (1) fragment size, (2) timing of measurements and (3) physiological proxies for heat tolerance. In experiment 2 (time-effect), ambient-treated corals experienced reductions in most physiological measures and thus responses were expressed as % change in physiological measures (colour change, *F*_*v*_/*F*_*m*_, chlorophyll-α and protein content, and catalase activity) in heated corals relative to their ambient counterparts.

### Experiment 1: Size effect

Nine colonies each of *A. tenuis* and *P. damicornis* (Table 3) from reef 13 to 123 were fragmented into six large (9.05 ± 0.44 cm^2^, 12.45 ± 0.7 cm^2^, *A. tenuis* and *P. damicornis*, respectively) and six small fragments (3.51 ± 0.19 cm^2^, 7.13 ± 0.34 cm^2^, *A. tenuis* and *P. damicornis*, respectively). Three large and three small fragments from each colony were assigned to the two treatments (ambient and + 6 °C, 1 size pair per tank per treatment, n = 216 for both species). The fragments were wrapped in aluminium foil and snap frozen in liquid N_2_ 11 h after the end of heat stress for further analysis.

### Experiment 2: Time effect

Samples of *A. tenuis* were collected from nine individual colonies at Creech reef (Table 3). Samples were further fragmented (18 per colony, ~ 5 cm in length), and distributed across treatments (ambient and + 6 °C, n = 3 fragments per colony per tank, total = 162 fragments). Sampling occurred immediately after the end of heat stress (T_0_), and then at 2 h (T_1_), 6 h (T_2_), 10 h (T_3_), 14 h (T_4_), 24 h (T_5_), and 48 h (T_6_) after the end of heat stress (Fig. 1C). At each sampling point, one fragment per colony per treatment was sampled apart from T_6_ (48 h) when all remaining fragments were sampled and preserved.

### Experiment 3: Alternative physiological measurements

Collections of *A. tenuis* to evaluate physiological metrics (including chlorophyll-*a* and protein content, catalase activity, tissue colour change, and photosynthetic efficiency) took place across five reefs and included 86 colonies (Table 3). Four fragments were made per colony except for reef 11-049 where only two fragments per colony were made. Fragments were distributed between treatments (n = 1 per colony per treatment) and sampled after an 11 h recovery period at ambient temperature.

### Photosynthetic efficiency

Photo-physiological status is a common diagnostic to measure effects of heat exposure and coral bleaching^8,35^ and thus we also quantified photosystem II (PSII) maximum photochemical efficiency (*F*_*v*_/*F*_*m*_, dimensionless) using Pulse Amplitude Modulated Fluorometry of chlorophyll-*a* (PAM, Diving-PAM, Heinz Walz GmbH, Effeltrich, Germany, MI = 8, SI = 8, saturation width = 0.8, Gain = 3, Damp = 2)^33,78^. A clear piece of PVC tubing was used to maintain a constant distance (2 mm) between the glass fibre-optic probe (6 mm Ø) and the coral fragment. Samples were dark acclimated for 30 min before measurements were taken. Each fragment was measured twice at different spots approx. 1/3 distance from the apical corallite. For experiments 1 (size) and 3 (physiological measures), values of *F*_*v*_/*F*_*m*_ were determined 10 h after the end of heat stress (Fig. 4B). For experiment 2 (time) *F*_*v*_/*F*_*m*_ was measured at sampling time points (T_0_–T_6_).

### Visual signs of bleaching

For experiments 1 (size) and 3 (physiological measures), samples were photographed prior to, and after, heat treatment with a digital SLR camera (Nikon D300, F stop = 4, shutter speed = 100, ISO = 400). For experiment 2 (time), samples were also photographed at each time point (T_0_–T_6_, Fig. 4C). Photographs were taken at a distance of 25 cm against a dark background, which included the *Coral Watch* colour reference chart^76^. Tissue colour was assessed as per Nielsen et al.^39^.

### Sampling and sample preparation for physiological assays

A pressurized air gun and 0.02 µm filtered seawater (FSW) was used to remove tissue from coral skeletons^79^. The resulting slurry was homogenised (30 s, 40% power, Pro200, Bio-gen Series, ProScientific, USA) and aliquots were removed for chlorophyll-*a* quantification (1 ml), centrifuged (5 min, 4 °C, 1500 rpm) and the supernatant discarded. The resulting symbiont pellet was stored dry at − 80 °C. Remaining tissue slurry was centrifuged (5 min, 4 °C, 1500 rpm) to separate host tissues from Symbiodiniaceae cells. Host tissue was aliquoted (500 µl) into 96-well tissue culture plates for protein analysis. For catalase activity, 1 ml of host tissue was aliquoted into Eppendorf tubes.

### Surface area quantification

Surface area of each blasted coral skeleton was quantified according to the double wax dipping method^80^, which has been shown to accurately calculate the surface area of branching species^81^*.* Skeletons were bleached (10%), rinsed, dried, and stored at room temperature prior to dipping. Cylindrical shapes of known sizes were used to produce a standard curve of surface area. Skeletons and standards were immersed (4 s) into hot wax (65 °C), removed, swirled to air-dry and left to dry for a further 15 min before weighing. The dipping procedure was repeated, and surface area calculated as the weight difference between the first and second dip.

### Chlorophyll-a quantification

Pre-chilled ethanol (0.8 ml, EtOH, 95%) was added to each frozen sample and vortexed until the pellet was fully resuspended then sonicated (3 min, 40% power, Sonic Power® MU-600, Mirae Ultrasonic Tech Co, Korea), vortexed, and incubated on ice in the dark to extract pigments (20 min). Triplicate aliquots (200 µl) were loaded onto a 96 microwell plate (Immulon® 4, HBX) using EtOH (95%) as a blank and absorbance was read immediately at 664 nm and 649 nm. Chlorophyll-*a* content was calculated following Eq. (1)^82,83^, corrected for absorbance in the blanks and normalised to surface area of the coral fragment.

1 $$(({13}.{36}*{\text{Abs}}_{{{664}\,{\text{nm}}}} ) - ({5}.{19}*{\text{Abs}}_{{{649}\,{\text{nm}}}} )){/}0.{794}$$

### Protein content

Water-soluble protein content was determined using the Bio-Rad *DC* Protein Assay following the manufacturer’s guidelines. Protein samples were thawed on ice, homogenised and diluted 1:1 in NaOH (200 µl, 1 M). Samples were sonicated using an ultrasound water bath for 5 min (40% amplitude, Sonic Power® MU-600, Mirae Ultrasonic Tech Co, Korea) before being digested in an oven for 1 h at 90 °C. Samples were then centrifuged (10 min, 2000 rpm) before loading 10 µl per replicate into a microtiter plate (96-well, 300 µl, Immulon® 4, HBX). Reagent A (25 µl) was added and allowed to stand for 5 min before adding 200 µl of Reagent B. The plate was covered and incubated in the dark at room temperature for 15 min. After incubation, the plate was loaded into the spectrophotometer (Synergy H4 Hybrid Reader®, Bio-Tek, Winooski, VT, USA) and absorbance read at 750 nm (25 °C). Protein content was normalised to surface area and reported as mg cm^−2^.

### Catalase activity

Catalase activity was quantified as the change in H_2_O_2_ concentration over time^31^. Samples were thawed on ice, vortexed (40 s) and 30 µl were added to a UV-transparent micro-well plate (UV-Star®, 96 wells, Greiner Bio-One, Monroe, NC, USA) in triplicate with FSW as blanks. 60 µl of PBS (50 mM, pH 7) and 20 µl of EDTA (10 mM) were added to the plate before adding 120 µl of H_2_O_2_ (50 mM) as substrate for the reaction. The plate was immediately loaded into the spectrophotometer (Synergy H4 Hybrid Reader®) and absorbance was read at 240 nm every 30 s for 15 min. Catalase activity (U) was assessed over the linear portion of the curve and expressed as specific activities (U mg^−1^ protein).

### Cost–benefit of alternative physiological measurements

A qualitative cost–benefit analysis was conducted to contrast the data returned relative to resource and time investment across the various physiological measurements. We identified the consumables and (capital) equipment required for each physiological measurement (Supplementary material S6). Cost of consumables per 100 samples was calculated from pricing available online or via direct quotes. No shipping or GST costs were included. The cost of equipment use per 100 samples was based on an approximation of how many samples were likely to be processed over a conservative lifespan of the respective item. Time estimates were based on our in-laboratory experiences processing the samples for this study (n = 779 fragments). Chlorophyll, protein, and catalase assays according to methods presented here, require the samples to be tissue blasted. Therefore, the cost and time requirement of tissue blasting should be accounted for if planning to conduct any of these. Similarly, these assays are standardised to fragment surface area, and the costs associated with this assay are therefore also included. No sample preservation costs were included in these estimates.

### Statistical analyses

All statistical analyses were conducted in R^84^. The effects of fragment size were investigated by generalised linear mixed effects models. We assumed a Gaussian distribution of all dependent variables and checked for normality of modelled residuals and homoscedasticity of plotted residuals (package *DHARMa*,^85^. The models were fitted by restricted maximum likelihood and generated using the glmmTMB function in the R package *glmmTMB*^86^, where treatment (ambient vs high) and fragment size (large vs small) were used as fixed effects. Colony identity was fitted as a random effect with a random intercept^87^. Model fit was evaluated by assumption fit and R^2,88^. Adjusted *p*-values for the Post-hoc Tukey HSD tests were calculated using the single-step method.

Because of the decline in coral condition in the ambient treatment evident in the time effect experiment, data were transformed to percent change in the heated treatment relative to ambient and modelled against a gaussian distribution using a linear mixed effects model using the *glmmTMB* R package^86^. Assumptions and homoscedasticity were confirmed as above. Time was modelled as a categorical variable rather than a continuous to allow direct, post-hoc comparisons between specific sampling times. Post-hoc comparisons were investigated with Tukey’s HSD tests.

The relative importance of multiple physiological metrics driving observed differences in thermal responses to acute heat stress was assessed by Principal Component Analysis (PCA) performed in R, using the package *vegan*^89^. Based on Eigenvalues (> 1), we used two principal components (PCs) to account for the variability within the data. PC1 (43%) and PC2 (21%) cumulatively accounted for 64% of the variance. Additionally, each physiological trait was correlated to each other and a heatmap produced using the *lattice* R package^90^.

## Supplementary Information

Supplementary Information 1.

Supplementary Information 2.

## Data Availability

Data and associated code to produce the statistical and graphical components of this manuscript are available on JJVN’s GitHub (https://github.com/josephinenielsen/AcuteHeatStressMethods_SciReps.git).
