# Supplementary material for: Experimental considerations of acute heat stress assays to quantify coral thermal tolerance
Source: Sci Rep. 2022 Oct 7;12:16831. doi: 10.1038/s41598-022-20138-2 (PMC9546840; doi:10.1038/s41598-022-20138-2)
Supplement: Supplementary file 1 — Supplementary Information 1. [file 41598_2022_20138_MOESM1_ESM.docx]

Experimental considerations of acute heat stress assays to quantify coral thermal tolerance

Supplementary Materials

Authors: JJV Nielsen*^1,2,3^, G Matthews^7^, KR Frith^2,8^, HB Harrison^2,4^, MR. Marzonie^2,3,4^, KL Slaughter^3,5^, DJ Suggett^6^, LK Bay^2^

S1 – Experimental tank system

The tank-based heat stress assay system was specifically designed for mobility and flexibility of application following the CBASS (Coral Bleaching Automated Stress System) principle outlined by Voolstra et al. (2020). Our system consisted of two sets of three tanks, one set with temperature control capability (temperature manipulation system, Fig.S1) and the other without (ambient system, Fig.S1). The temperature manipulation system consisted of three independent acrylic tanks (55L), each supplied with heated flow-through seawater (55L h^-1^). Tanks were placed in water jackets to aid in temperature control and stability. The jackets were supplied with recirculating, warm seawater, heated with a titanium heating element (Omega 2kW) held in a separate jacket (sump, Fig.S1) and pumped between jackets using a submersible pump (Reefe RP2400LV 24v). The sump also held a heat exchange coil (Wateco 56”) to heat seawater delivered to the tanks. Temperature was controlled with a programmable logic controller (Siemens S7 15-11-1 PN). For the ambient system, both the jackets and experimental tanks were supplied with flow-through seawater pumped from the ocean (55L h^-1^). Every tank was fitted with a circulation pump (Turbelle® nanostream® 6055, Tunze, Penzburg, Germany). The entire system was set-up in the wet laboratory of the *RV Solander* and supplied with artificial lights (Hydra, AquaIllumination®, 400-700nm, C2 Development, Ames, Iowa, USA; 450 µmol photons m^-2^ s^-1^, no ramping, 7h:11h light:dark, 60% blue, 20% white, 10% green, and 10% red) suspended 40 cm above each tank. An initial light intensity of 550 µmol photons m^-2^ s^-1^ was chosen based on the light profile of the region for summer (AIMS light data [Lizard Island Light From 26 Feb 2012 | AIMS metadata | aims.gov.au](https://apps.aims.gov.au/metadata/view/cb577ca6-7098-4a53-b9aa-fea076bcaa55)); however, this intensity led to visible bleaching in the ambient-treated corals and so was reduced for subsequent experimentation.


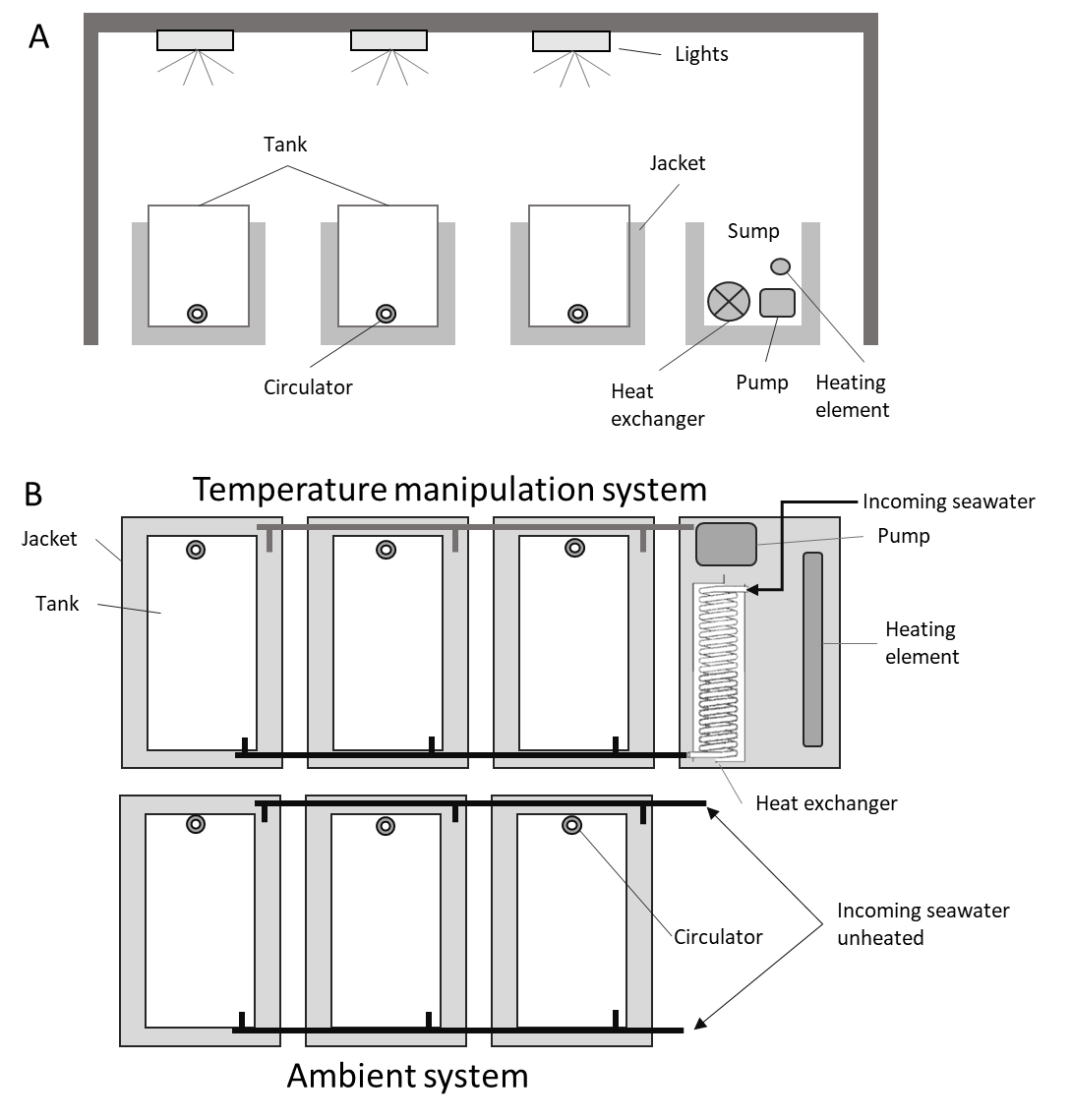


Fig S1 Schematic overview of the experimental tank set-up. (A) Side view of the temperature manipulation system, with three experimental tanks in water jackets and a fourth jacket acting as sump for heating water for both tanks and jackets. The sump contained the heating element, heat exchange coil, and a submersible pump to pump recirculating water to the jackets while the tanks were supplied with warm water from the heating coil. (B) Top-down view of the temperature manipulation and ambient systems. The ambient system was supplied unheated seawater to both jackets and tanks.

S2 - All experimental temperatures

Table S2 Treatment temperature (High and Mid) achieved during each acute heat stress experimental run compared to the ambient sea-water temperature.

| **Experiment** | **Reef** | **Treatment combo** | **Mean heated temperature** | **Heated SE** | **Mean ambient temperature** | **Ambient SE** | **Temperature difference** |
| --- | --- | --- | --- | --- | --- | --- | --- |
| 1 | 13-123  A. tenuis | High/Ambient | 34.89 | 0.059 | 29.94 | 0.026 | 4.952381 |
|  | 13-123  P. damicornis | High/Ambient | 35.02 | 0.068 | 30.03 | 0.049 | 4.990476 |
| 2 | Creech | High/Ambient | 34.63 | 0.097 | 29.59 | 0.029 | 5.038889 |
| 3 | 11-049 | High/Ambient | 34.99 | 0.12 | 29.96 | 0.020 | 5.035714 |
|  | 13-124 | High/Ambient | 34.98 | 0.061 | 30.55 | 0.013128 | 4.433333 |
|  | 13-124 | Mid/Ambient | 33.44 | 0.027 | 30.2 | 0.040237 | 3.24 |
|  | Corbett | High/Ambient | 35.23 | 0.037 | 30.8 | 0.016903 | 4.433333 |
|  | Corbett | Mid/Ambient | 32.99 | 0.060 | 30.39 | 0.015065 | 2.594444 |
|  | Davie | High/Ambient | 34.99 | 0.034 | 30.87 | 0.062994 | 4.12 |
|  | Davie | Mid/Ambient | 34.21 | 0.031 | 29.96 | 0.066898 | 4.255556 |
|  | Lagoon | High/Ambient | 35.09 | 0.089 | 29.53 | 0.023035 | 5.561905 |
|  | Lagoon | Mid/Ambient | 33.37 | 0.049 | 29.46 | 0.013436 | 3.911111 |
|  | Mantis | High/Ambient | 35.28 | 0.085 | 30.37 | 0.027021 | 4.913333 |
|  | Mantis | Mid/Ambient | 33.53 | 0.079 | 29.84 | 0.082896 | 3.688889 |


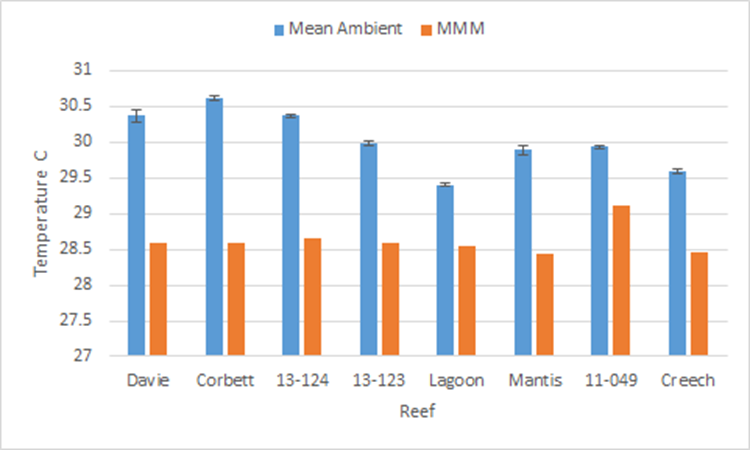


Fig S2. Difference between mean (±SE) ambient temperature at time of experiment and the reef-specific Max Monthly Mean (MMM) temperature obtained from NOAA.

S3 – Statistical outputs for size effects in *A. tenuis* and *P. damicornis*

Table S3.1. Statistical outputs for size and treatment effects in *A. tenuis* and *P. damicornis*.

| Species | Trait | Transformation | Term | df | Z | p |
| --- | --- | --- | --- | --- | --- | --- |
| *A. tenuis* | Colour change | Cube root | Treatment | 106 | -6.714 | 1.89E-11 |
|  |  |  | Size | 106 | 4.231 | 2.32E-05 |
|  |  |  | Treatment*Size | 106 | -3.255 | 0.00114 |
|  | Chlorophyll *a* | NA | Treatment | 104 | -6.236 | < 0.0001 |
|  |  |  | Size | 104 | 1.19 | 0.234 |
|  |  |  | Treatment*Size | 104 | -0.079 | 0.937 |
|  | Catalase activity | log | Treatment | 93 | 2.382 | 0.0172 |
|  |  |  | Size | 93 | -1.309 | 0.1904 |
|  |  |  | Treatment*Size | 93 | -1.281 | 0.2003 |
|  | Protein content | log | Treatment | 91 | -5.112 | < 0.0001 |
|  |  |  | Size | 91 | -0.775 | 0.438 |
|  |  |  | Treatment*Size | 91 | 0.882 | 0.378 |
|  | *F_v_*/*F_m_* | NA | Treatment | 212 | -10.13 | < 0.0001 |
|  |  |  | Size | 212 | 0.82 | 0.413 |
|  |  |  | Treatment*Size | 212 | 1.26 | 0.207 |
| *P. damicornis* | Colour change | Cube root | Treatment | 106 | -9.273 | < 0.0001 |
|  |  |  | Size | 106 | -2.631 | 0.0085 |
|  |  |  | Treatment*Size | 106 | 2.499 | 0.0125 |
|  | Chlorophyll *a* | NA | Treatment | 96 | -2.776 | 0.0055 |
|  |  |  | Size | 96 | 2.653 | 0.00797 |
|  |  |  | Treatment*Size | 96 | -1.975 | 0.04827 |
|  | Catalase activity | log | Treatment | 86 | -4.546 | < 0.0001 |
|  |  |  | Size | 86 | -1.851 | 0.064175 |
|  |  |  | Treatment*Size | 86 | 2.82 | 0.004804 |
|  | Protein content | log | Treatment | 102 | -3.173 | 0.00151 |
|  |  |  | Size | 102 | -2.761 | 0.00577 |
|  |  |  | Treatment*Size | 102 | 0.136 | 0.89186 |
|  | *F_v_*/*F_m_* | NA | Treatment | 195 | -8.15 | < 0.0001 |
|  |  |  | Size | 195 | -1.31 | 0.192 |
|  |  |  | Treatment*Size | 195 | 0.27 | 0.785 |

Table S3.2 Post-hoc contrasts of physiological traits for both *A. tenuis* and *P. damicornis*.

| Species | Trait | Contrast | df | T ratio | p |
| --- | --- | --- | --- | --- | --- |
| *A. tenuis* | Colour change | Large Heated - Large Ambient | 106 | 6.714 | < 0.0001 |
|  |  | Small Ambient - Large Ambient | 106 | -4.231 | < 0.0001 |
|  |  | Small Heated - Large Heated | 106 | 0.371 | 0.7114 |
|  |  | Small Heated - Small Ambient | 106 | 11.316 | < 0.0001 |
| *P. damicornis* | Chlorophyll *a* | Large Heated - Large Ambient | 96 | 2.776 | 0.0066 |
|  |  | Small Ambient - Large Ambient | 96 | 5.586 | < 0.0001 |
|  |  | Small Heated - Large Heated | 96 | -2.653 | 0.0093 |
|  |  | Small Heated - Small Ambient | 96 | 0.177 | 0.8603 |
|  | Colour change | Large Heated - Large Ambient | 106 | 9.279 | < 0.0001 |
|  |  | Small Ambient - Large Ambient | 106 | 5.745 | < 0.0001 |
|  |  | Small Heated - Large Heated | 106 | 2.631 | 0.0098 |
|  |  | Small Heated - Small Ambient | 106 | -0.902 | 0.3689 |
|  | Catalase activity | Large Heated - Large Ambient | 86 | 4.546 | < 0.0001 |
|  |  | Small Ambient - Large Ambient | 86 | 0.225 | 0.8228 |
|  |  | Small Heated - Large Heated | 86 | 1.851 | 0.0676 |
|  |  | Small Heated - Small Ambient | 86 | -2.146 | 0.0347 |

S4 – Statistical outputs from time effect analysis

Table S4.1 Statistical outputs for sampling time effect in *A. tenuis*.

| Trait | Term | estimate | d. error | z | p |
| --- | --- | --- | --- | --- | --- |
| Catalase | Intercept | -9.22 | 26.014 | -0.35 | 0.72 |
|  | T_1_ | -56.85 | 33.85 | -1.68 | 0.093 |
|  | T_2_ | 109.48 | 33.8 | 3.24 | 0.0012 |
|  | T_3_ | 40.42 | 34.34 | 1.18 | 0.24 |
|  | T_4_ | -34.53 | 34.56 | -1.00 | 0.32 |
|  | T_5_ | -88.38 | 35.40 | -2.50 | 0.013 |
|  | T_6_ | -74.58 | 32.88 | -2.27 | 0.023 |
| Chlorophyll | Intercept | -24.18 | 9.90 | -2.44 | 0.015 |
|  | T_1_ | -17.29 | 14.57 | -1.19 | 0.24 |
|  | T_2_ | -27.73 | 13.55 | -2.05 | 0.041 |
|  | T_3_ | -41.19 | 13.20 | -3.12 | 0.0018 |
|  | T_4_ | -35.25 | 13.55 | -2.60 | 0.0093 |
|  | T_5_ | -59.65 | 14.00 | -4.26 | < 0.0001 |
|  | T_6_ | -54.06 | 13.20 | -4.10 | < 0.0001 |
| Colour change | Intercept | -9.98 | 3.14 | -3.18 | 0.0015 |
|  | T_1_ | -4.49 | 2.93 | -1.53 | 0.13 |
|  | T_2_ | -8.96 | 2.93 | -3.05 | 0.0023 |
|  | T_3_ | -15.33 | 2.93 | -5.23 | < 0.0001 |
|  | T_4_ | -24.73 | 2.93 | -8.43 | < 0.0001 |
|  | T_5_ | -30.70 | 2.93 | -10.46 | < 0.0001 |
|  | T_6_ | -37.81 | 2.93 | -12.89 | < 0.0001 |
| Protein | Intercept | -26.44 | 11.25 | -2.35 | 0.019 |
|  | T_1_ | -10.89 | 16.56 | -0.66 | 0.51 |
|  | T_2_ | -24.05 | 16.91 | -1.51 | 0.13 |
|  | T_3_ | -57.61 | 16.56 | -3.48 | 0.0005 |
|  | T_4_ | -58.08 | 15.91 | -3.65 | 0.0003 |
|  | T_5_ | -73.56 | 15.91 | -4.62 | < 0.0001 |
|  | T_6_ | -73.56 | 15.41 | -4.78 | < 0.0001 |
| F_v_/F_m_ | Intercept | -5.85 | 2.43 | -2.40 | 0.016 |
|  | T_1_ | -3.85 | 3.01 | -1.28 | 0.20 |
|  | T_2_ | -1.61 | 3.01 | -0.54 | 0.59 |
|  | T_3_ | 0.60 | 3.01 | 0.20 | 0.84 |
|  | T_4_ | 0.40 | 3.01 | 0.13 | 0.90 |
|  | T_5_ | -4.43 | 3.11 | -1.43 | 0.154 |
|  | T_6_ | -32.10 | 3.01 | -10.65 | < 0.0001 |

Table S4.2 Post-hoc contrasts of physiological traits over time, *A. tenuis*.

| Traits | contrast | estimate | SE | df | t ratio | p value |
| --- | --- | --- | --- | --- | --- | --- |
| Protein | T0 - T1 | 10.88921 | 16.6 | 39 | 0.658 | 0.9942 |
|  | T0 - T2 | 24.04836 | 15.9 | 39 | 1.511 | 0.7364 |
|  | T0 - T3 | 57.61369 | 16.6 | 39 | 3.479 | **0.0196** |
|  | T0 - T4 | 58.07697 | 15.9 | 39 | 3.65 | **0.0124** |
|  | T0 - T5 | 73.55848 | 15.9 | 39 | 4.623 | **0.0008** |
|  | T0 - T6 | 73.55836 | 15.4 | 39 | 4.775 | **0.0005** |
|  | T1 - T2 | 13.15916 | 16.6 | 39 | 0.795 | 0.9842 |
|  | T1 - T3 | 46.72448 | 17.2 | 39 | 2.719 | 0.1204 |
|  | T1 - T4 | 47.18777 | 16.6 | 39 | 2.849 | 0.0908 |
|  | T1 - T5 | 62.66928 | 16.6 | 39 | 3.784 | **0.0086** |
|  | T1 - T6 | 62.66915 | 16.1 | 39 | 3.898 | **0.0063** |
|  | T2 - T3 | 33.56532 | 16.6 | 39 | 2.027 | 0.415 |
|  | T2 - T4 | 34.02861 | 15.9 | 39 | 2.139 | 0.3513 |
|  | T2 - T5 | 49.51012 | 15.9 | 39 | 3.112 | **0.0495** |
|  | T2 - T6 | 49.50999 | 15.4 | 39 | 3.214 | **0.0386** |
|  | T3 - T4 | 0.46329 | 16.6 | 39 | 0.028 | 1 |
|  | T3 - T5 | 15.9448 | 16.6 | 39 | 0.963 | 0.959 |
|  | T3 - T6 | 15.94467 | 16.1 | 39 | 0.992 | 0.9528 |
|  | T4 - T5 | 15.48151 | 15.9 | 39 | 0.973 | 0.9569 |
|  | T4 - T6 | 15.48138 | 15.4 | 39 | 1.005 | 0.9498 |
|  | T5 - T6 | -0.00012 | 15.4 | 39 | 0 | 1 |
| Chlorophyll | T0 - T1 | 17.29 | 14.6 | 45 | 1.187 | 0.8955 |
|  | T0 - T2 | 27.73 | 13.6 | 45 | 2.046 | 0.4014 |
|  | T0 - T3 | 41.19 | 13.2 | 45 | 3.121 | **0.0457** |
|  | T0 - T4 | 35.24 | 13.6 | 45 | 2.601 | 0.15 |
|  | T0 - T5 | 59.65 | 14 | 45 | 4.262 | **0.0018** |
|  | T0 - T6 | 54.06 | 13.2 | 45 | 4.097 | **0.0031** |
|  | T1 - T2 | 10.44 | 14.1 | 45 | 0.738 | 0.9893 |
|  | T1 - T3 | 23.9 | 13.8 | 45 | 1.732 | 0.599 |
|  | T1 - T4 | 17.96 | 14.1 | 45 | 1.27 | 0.8618 |
|  | T1 - T5 | 42.36 | 14.6 | 45 | 2.908 | 0.0764 |
|  | T1 - T6 | 36.77 | 13.8 | 45 | 2.665 | 0.1313 |
|  | T2 - T3 | 13.46 | 12.7 | 45 | 1.058 | 0.9369 |
|  | T2 - T4 | 7.52 | 13.1 | 45 | 0.574 | 0.9972 |
|  | T2 - T5 | 31.92 | 13.6 | 45 | 2.356 | 0.2412 |
|  | T2 - T6 | 26.33 | 12.7 | 45 | 2.07 | 0.3876 |
|  | T3 - T4 | -5.94 | 12.7 | 45 | -0.467 | 0.9991 |
|  | T3 - T5 | 18.46 | 13.2 | 45 | 1.399 | 0.7993 |
|  | T3 - T6 | 12.87 | 12.3 | 45 | 1.043 | 0.941 |
|  | T4 - T5 | 24.4 | 13.6 | 45 | 1.801 | 0.5544 |
|  | T4 - T6 | 18.81 | 12.7 | 45 | 1.479 | 0.7556 |
|  | T5 - T6 | -5.59 | 13.2 | 45 | -0.424 | 0.9995 |
|  |  |  |  |  |  |  |
| Traits | **contrast** | **estimate** | **SE** | **df** | **t ratio** | **p value** |
| Colour change | T0 - T1 | 4.49 | 2.93 | 53 | 1.532 | 0.7249 |
|  | T0 - T2 | 8.96 | 2.93 | 53 | 3.054 | 0.0513 |
|  | T0 - T3 | 15.33 | 2.93 | 53 | 5.225 | **0.0001** |
|  | T0 - T4 | 24.73 | 2.93 | 53 | 8.429 | **<.0001** |
|  | T0 - T5 | 30.7 | 2.93 | 53 | 10.462 | **<.0001** |
|  | T0 - T6 | 37.81 | 2.93 | 53 | 12.887 | **<.0001** |
|  | T1 - T2 | 4.47 | 2.83 | 53 | 1.577 | 0.6971 |
|  | T1 - T3 | 10.84 | 2.83 | 53 | 3.826 | **0.006** |
|  | T1 - T4 | 20.24 | 2.83 | 53 | 7.145 | **<.0001** |
|  | T1 - T5 | 26.2 | 2.83 | 53 | 9.251 | **<.0001** |
|  | T1 - T6 | 33.32 | 2.83 | 53 | 11.763 | **<.0001** |
|  | T2 - T3 | 6.37 | 2.83 | 53 | 2.249 | 0.2878 |
|  | T2 - T4 | 15.77 | 2.83 | 53 | 5.568 | **<.0001** |
|  | T2 - T5 | 21.74 | 2.83 | 53 | 7.674 | **<.0001** |
|  | T2 - T6 | 28.85 | 2.83 | 53 | 10.186 | **<.0001** |
|  | T3 - T4 | 9.4 | 2.83 | 53 | 3.318 | 0.0257 |
|  | T3 - T5 | 15.37 | 2.83 | 53 | 5.425 | **<.0001** |
|  | T3 - T6 | 22.48 | 2.83 | 53 | 7.937 | **<.0001** |
|  | T4 - T5 | 5.97 | 2.83 | 53 | 2.106 | 0.3641 |
|  | T4 - T6 | 13.08 | 2.83 | 53 | 4.619 | **0.0005** |
|  | T5 - T6 | 7.12 | 2.83 | 53 | 2.512 | 0.1758 |
| Fv/Fm | T0 - T1 | 3.849 | 3.01 | 51 | 1.277 | 0.8591 |
|  | T0 - T2 | 1.612 | 3.01 | 51 | 0.535 | 0.9982 |
|  | T0 - T3 | -0.597 | 3.01 | 51 | -0.198 | 1 |
|  | T0 - T4 | -0.396 | 3.01 | 51 | -0.131 | 1 |
|  | T0 - T5 | 4.428 | 3.11 | 51 | 1.426 | 0.7855 |
|  | T0 - T6 | 32.099 | 3.01 | 51 | 10.651 | **<.0001** |
|  | T1 - T2 | -2.237 | 2.8 | 51 | -0.798 | 0.9841 |
|  | T1 - T3 | -4.446 | 2.8 | 51 | -1.586 | 0.6916 |
|  | T1 - T4 | -4.245 | 2.8 | 51 | -1.514 | 0.735 |
|  | T1 - T5 | 0.579 | 2.9 | 51 | 0.2 | 1 |
|  | T1 - T6 | 28.25 | 2.8 | 51 | 10.078 | **<.0001** |
|  | T2 - T3 | -2.209 | 2.8 | 51 | -0.788 | 0.9851 |
|  | T2 - T4 | -2.008 | 2.8 | 51 | -0.716 | 0.991 |
|  | T2 - T5 | 2.816 | 2.9 | 51 | 0.972 | 0.9578 |
|  | T2 - T6 | 30.488 | 2.8 | 51 | 10.876 | **<.0001** |
|  | T3 - T4 | 0.201 | 2.8 | 51 | 0.072 | 1 |
|  | T3 - T5 | 5.025 | 2.9 | 51 | 1.734 | 0.5969 |
|  | T3 - T6 | 32.696 | 2.8 | 51 | 11.664 | **<.0001** |
|  | T4 - T5 | 4.824 | 2.9 | 51 | 1.665 | 0.6417 |
|  | T4 - T6 | 32.496 | 2.8 | 51 | 11.592 | **<.0001** |
|  | T5 - T6 | 27.672 | 2.9 | 51 | 9.551 | **<.0001** |
|  |  |  |  |  |  |  |
| Traits | contrast | estimate | SE | df | t ratio | p value |
| Catalase | T0 - T1 | 56.8 | 33.8 | 37 | 1.679 | 0.6334 |
|  | T0 - T2 | -109.5 | 33.8 | 37 | -3.239 | **0.0371** |
|  | T0 - T3 | -40.4 | 34.3 | 37 | -1.177 | 0.8983 |
|  | T0 - T4 | 34.5 | 34.6 | 37 | 0.999 | 0.951 |
|  | T0 - T5 | 88.4 | 35.4 | 37 | 2.497 | 0.1899 |
|  | T0 - T6 | 74.6 | 32.9 | 37 | 2.268 | 0.2861 |
|  | T1 - T2 | -166.3 | 33.8 | 37 | -4.921 | **0.0003** |
|  | T1 - T3 | -97.3 | 34.3 | 37 | -2.832 | 0.0956 |
|  | T1 - T4 | -22.3 | 34.6 | 37 | -0.646 | 0.9947 |
|  | T1 - T5 | 31.5 | 35.4 | 37 | 0.891 | 0.9717 |
|  | T1 - T6 | 17.7 | 32.9 | 37 | 0.539 | 0.998 |
|  | T2 - T3 | 69.1 | 33.1 | 37 | 2.089 | 0.38 |
|  | T2 - T4 | 144 | 31.9 | 37 | 4.51 | **0.0011** |
|  | T2 - T5 | 197.9 | 33 | 37 | 6.002 | **<.0001** |
|  | T2 - T6 | 184.1 | 30.6 | 37 | 6.006 | **<.0001** |
|  | T3 - T4 | 74.9 | 33.7 | 37 | 2.223 | 0.3083 |
|  | T3 - T5 | 128.8 | 34.6 | 37 | 3.726 | **0.0105** |
|  | T3 - T6 | 115 | 32.3 | 37 | 3.562 | **0.0163** |
|  | T4 - T5 | 53.9 | 33.1 | 37 | 1.629 | 0.6649 |
|  | T4 - T6 | 40.1 | 30.5 | 37 | 1.312 | 0.842 |
|  | T5 - T6 | -13.8 | 31.9 | 37 | -0.433 | 0.9994 |

S5 – PCA support graphs


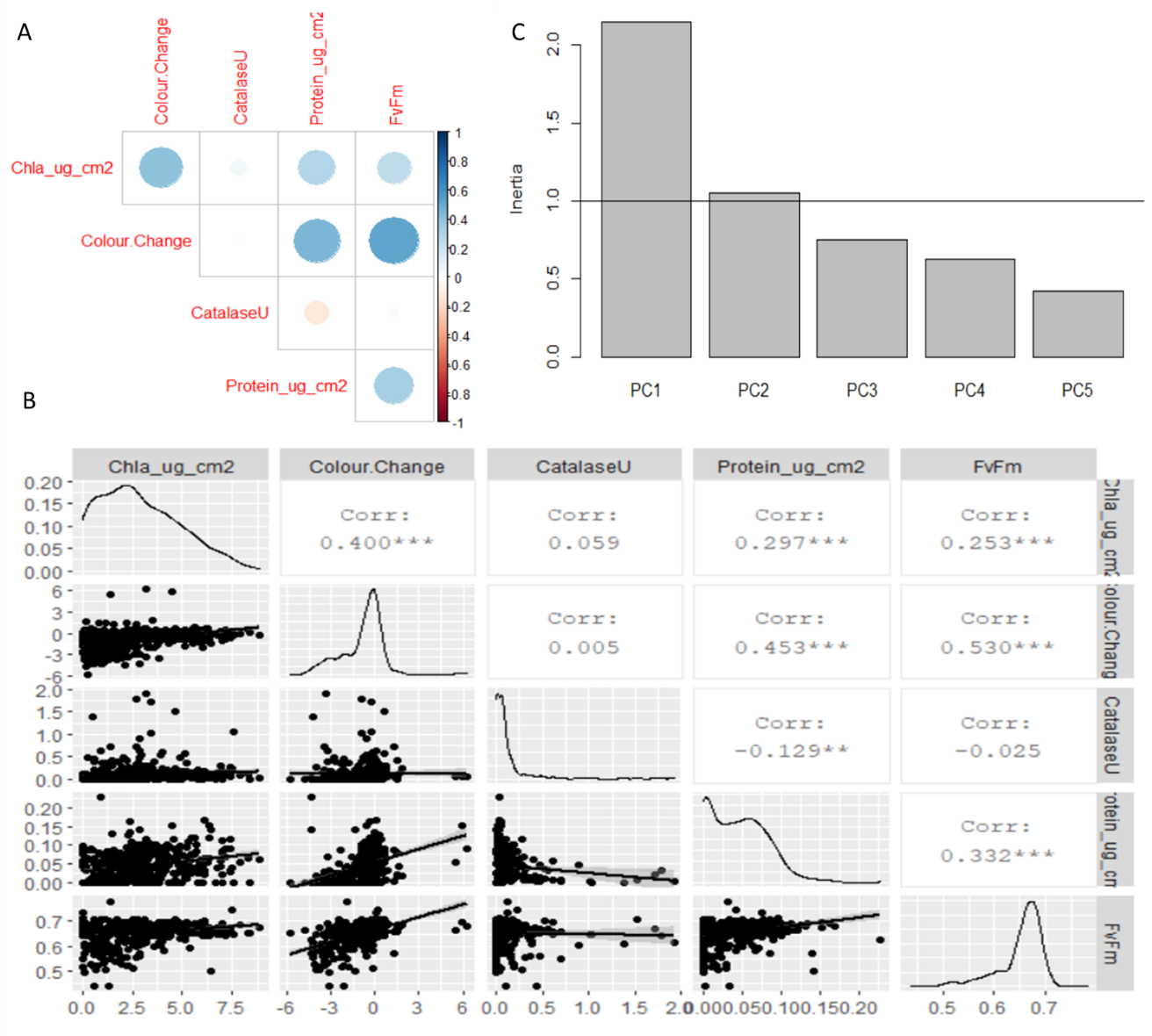


Fig S5. Support graphs for Principal Component Analysis of alternative physiological measurements. (A and B); some of the physiological measurements were correlated to each other. C; Screeplot showing the Eigen values of the five principle components generated. Principle components were plotted only for those where inertia > 1 (PC1 and PC2, specifically).

S6 – Cost benefit analysis details

The cost of equipment use per 100 samples was based on an approximation of how many samples were likely to be processed over a conservative lifespan of the respective item. For example, we assumed that a refrigerated centrifuge would have a lifespan of at least 10,000 samples whereas an icebox would only last for 1,000 samples (see supp mat). However, within this calculation we did not consider differences in centrifuge times across different physiological measurements, where the centrifuge is used once for chlorophyll extractions as opposed to three times successively for tissue blasting.

Table S6.1 Capital cost and lifespan of equipment required.

| **Equipment** | **Assays required for** | **Cost** | **Lifespan (# samples)** |
| --- | --- | --- | --- |
| Diving-PAM | Photosynthesis efficiency | $49,074 | 100,000 |
| Camera + memory card | Tissue colour change | $1,800 | 100,000 |
| Memory card (SD) | Tissue colour change | $55 | 100,000 |
| Coral Health Colour Chart | Tissue colour change | $5 | 1,000 |
| Airgun | Tissue blasting | $63.72 | 10,000 |
| Centrifuge, refrigerated | Tissue blasting, chlorophyll, protein | $11,720 | 10,000 |
| Styrofoam coolers | Tissue blasting, chlorophyll, protein, catalase | $10 | 1,000 |
| Homogeniser | Tissue blasting | $927 | 10,000 |
| Pipette, single channel | Tissue blasting, chlorophyll, protein, catalase, symbiont density | $432 | 10,000 |
| Vortex | Tissue blasting, chlorophyll, catalase, symbiont density | $369 | 10,000 |
| Oven | Protein | $2,220 | 100,000 |
| Pipette, multi-channel | Protein, catalase | $1,420 | 10,000 |
| Sonicator | Chlorophyll, protein | $1,500 | 10,000 |
| Spectrophotometer | Chlorophyll, protein, catalase | $25,000 | 100,000 |
| Stopwatch | Chlorophyll, protein, catalase, surface area | $12 | 1,000 |
| Scale | Surface area, chemical preparations | $995 | 10,000 |
| Forceps | Tissue blasting, surface area | $1.58 | 1,000 |
| Waterbath | Surface area | $1,115 | 10,000 |
| Ultralow freezer | Sample storage | $50,000 | 100,000 |

Table S6.2 Overview of other costs, special consideration, and benefits for each assay examined here.

| **Assay** | **Other costs/special considerations** | **Benefit** |
| --- | --- | --- |
| Photosynthetic efficiency | Expensive initial outlay for instrument | In-field data gathering |
|  |  | Range of photo-physiological data available |
| Tissue colour change |  | In-field data gathering |
|  |  | Rapid processing - especially with more automation coming online |
|  |  | Accessible technology |
| Tissue blasting | Samples require special storage to be viable | Leg-work for a wide range of physiological measurements |
|  | Requires multiple pieces of laboratory equipment |  |
| Chlorophyll | Ethanol (hazardous chemical) or other solvent | Specific measurement of symbiont bleaching response |
|  | Specialist training - spectrophotometer |  |
| Protein | Specialist training - spectrophotometer | Specific measurement of either symbiont and/or host physiological response to heat stress |
|  | Requires extraction kits |  |
| Catalase | Long downstream data processing | Specific measurement of either symbiont and/or host physiological response to heat stress |
|  | Expensive microwell plates required |  |
|  | Specialist training - spectrophotometer |  |
| Surface area | Required for most assays listed | Cheaper than 3D photogrammetry methods |
|  | Prone to operator error |  |
|  | Less accurate than 3D photogrammetry methods |  |
