# Supplementary material for: Experimental considerations of acute heat stress assays to quantify coral thermal tolerance
Source: Sci Rep. 2022 Oct 7;12:16831. doi: 10.1038/s41598-022-20138-2 (PMC9546840; doi:10.1038/s41598-022-20138-2)

Pricing for Cost Benefit Analysis in Nielsen et al Acute Heat Assays Methods paper

**Ethanol – absolute, 2.5L**

[Ethanol | Sigma-Aldrich (sigmaaldrich.com)](https://www.sigmaaldrich.com/AU/en/search/ethanol?focus=products&page=1&perPage=30&sort=relevance&term=Ethanol&type=product)


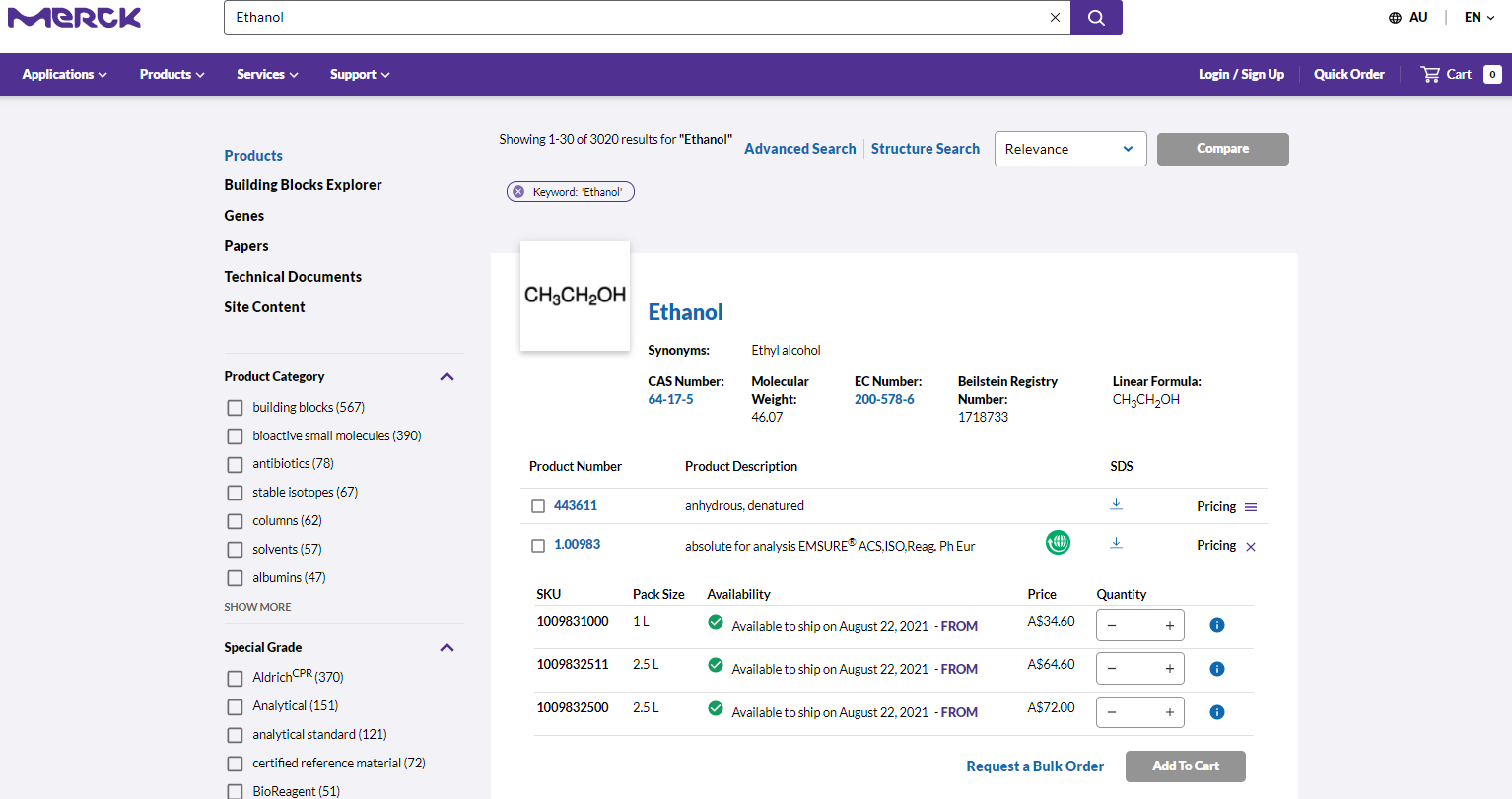


**Pipette tips – red, green, and blue used**

[SpaceSaver Pipette Tip Refills | Made from recycled PETE (mt.com)](https://www.mt.com/au/en/home/products/pipettes/bioclean-pipette-tip/packaging/spacesaver.html)


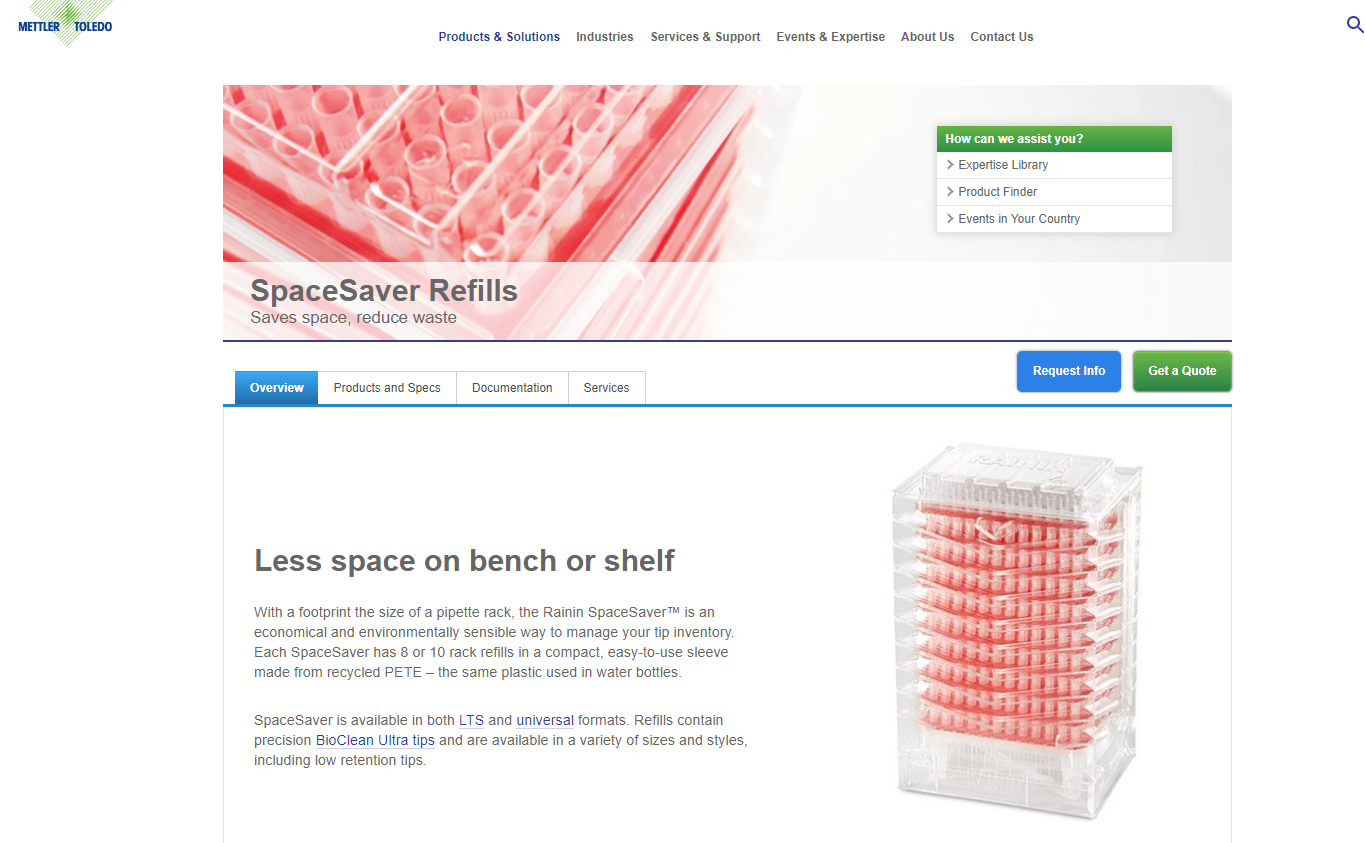


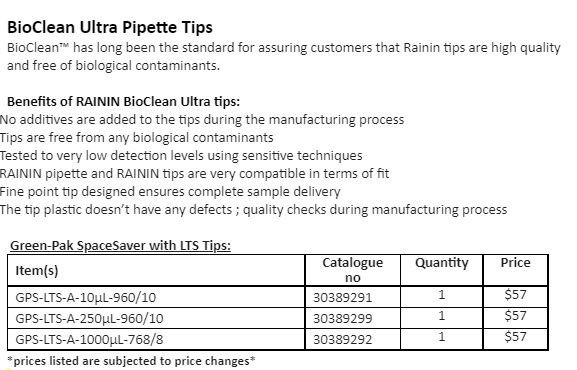


**Spec plates – Chlorophyll and protein assays only**

[Immulon® Immunoassay Plates and Strip Assemblies | Krackeler Scientific, Inc.](https://www.krackeler.com/catalog/product/2613/Immulon-Immunoassay-Plates-and-Strip-Assemblies)


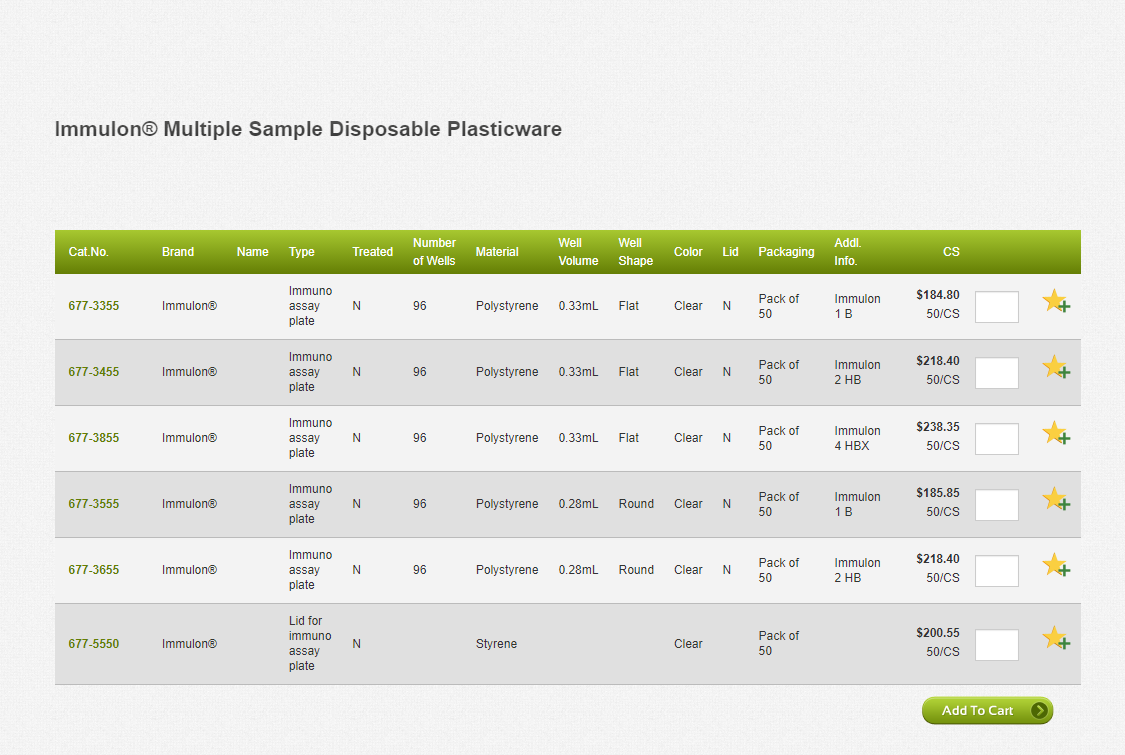


Deep well plates

[SSI Deep Well Plate - 2.0 mL, 96-well, Square, V Bottom (5/pack) | LabGear Australia - Laboratory Equipment and Consumables for the Australian Scientific and Research Community](https://www.labgearaustralia.com.au/shop/product/ssi-deep-well-plate-2-0-ml-96-well-square-v-bottom-5-pack-5589?category=154)


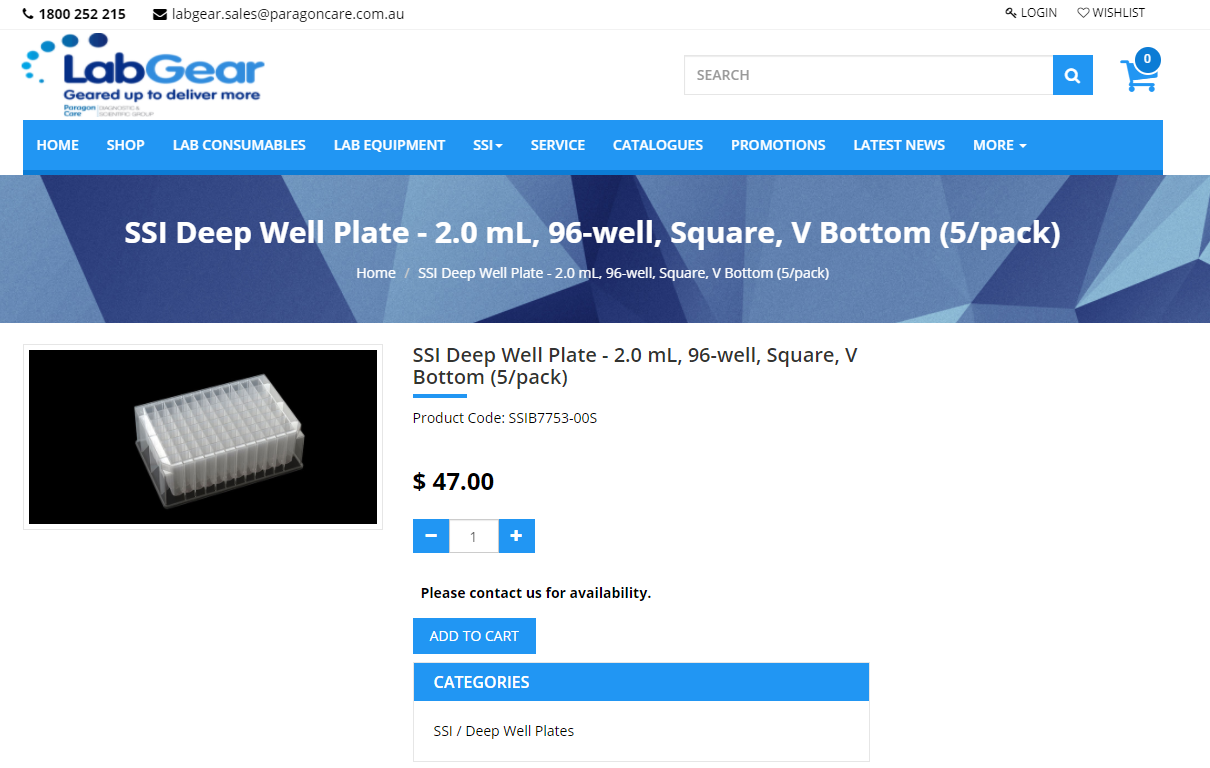


**Air blow gun**

[MettleAir AG2-100 4" Compressed Air Blow Gun, 1/4" NPT, Inlet Commercial Grade, AG2-100 (Pack of 10): Amazon.com: Tools & Home Improvement](https://www.amazon.com/mettleair-ag2-100-compressed-inlet-commercial/dp/b00s511j2o)


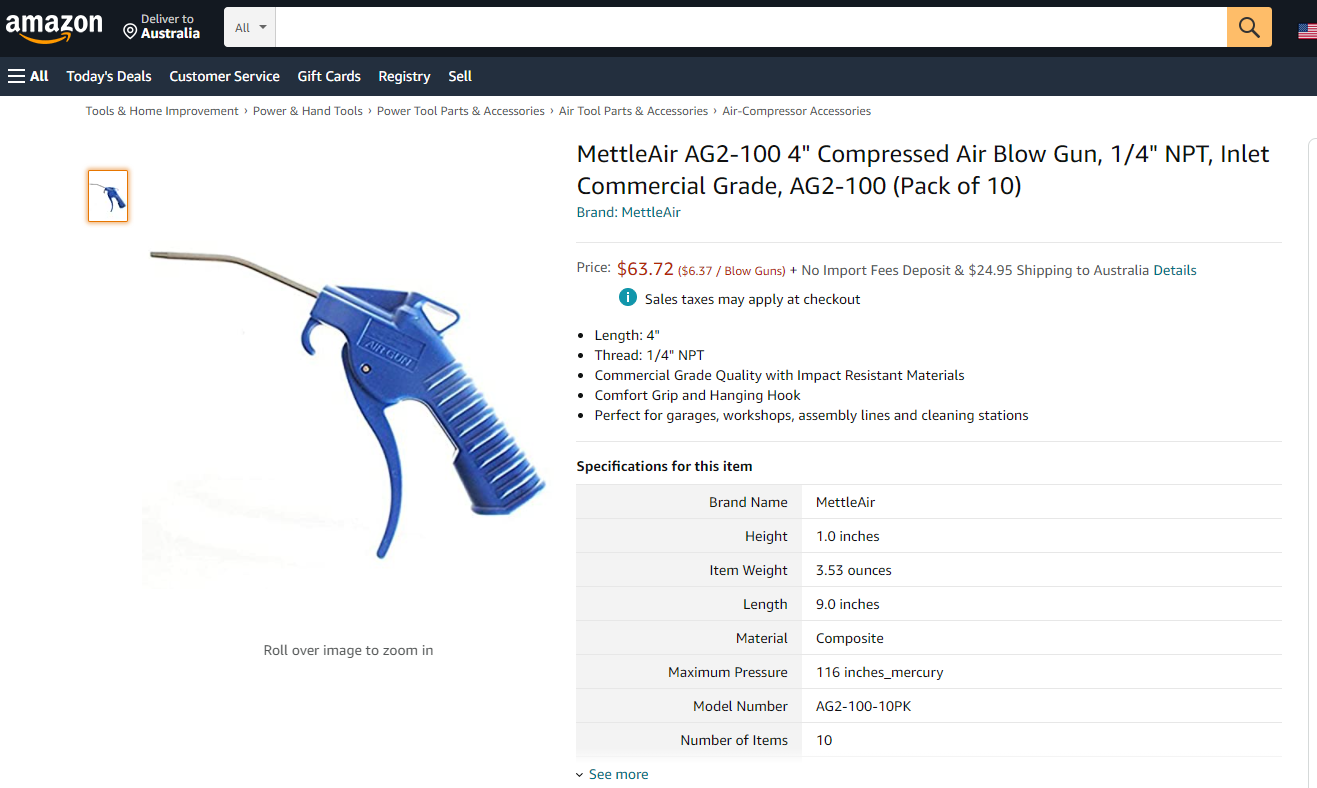


**Aluminium foil**


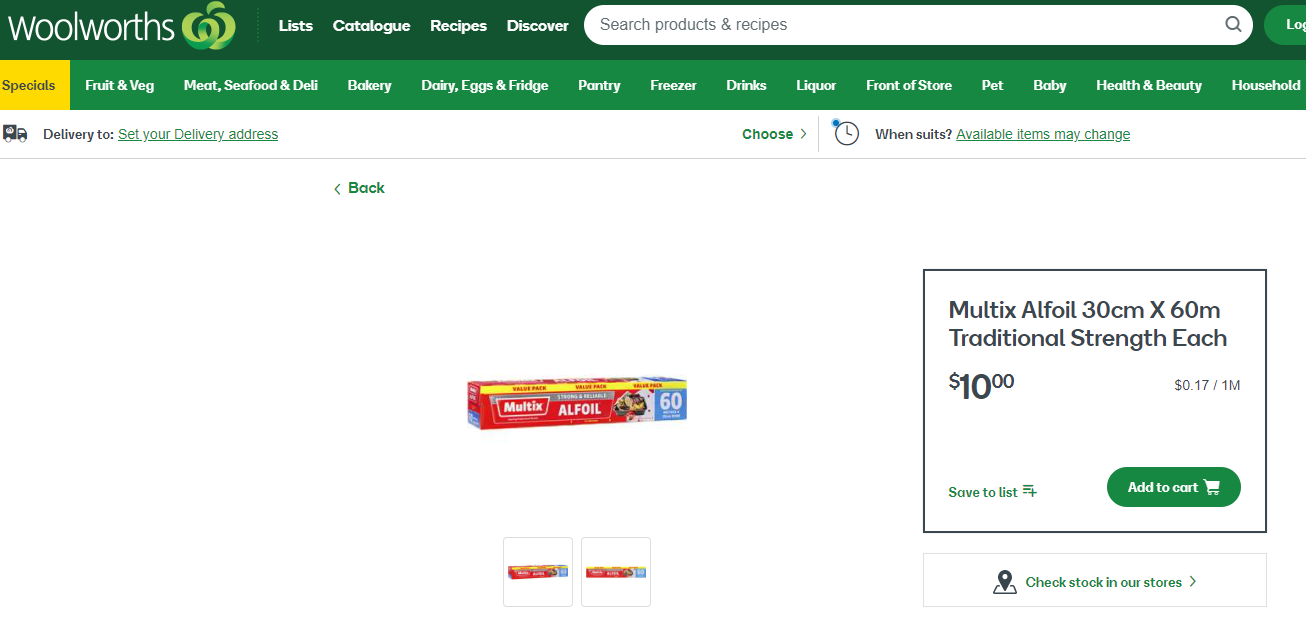


**Refrigerated centrifuge with falcon-tube capacity**

[Centrifuge 5804/ 5804 R - Multipurpose Centrifuges, Centrifugation - Eppendorf South Pacific](https://online-shop.eppendorf.com.au/AU-en/Centrifugation-44533/Multipurpose-Centrifuges-1007184/Centrifuge-5804-5804R-PF-240993.html)


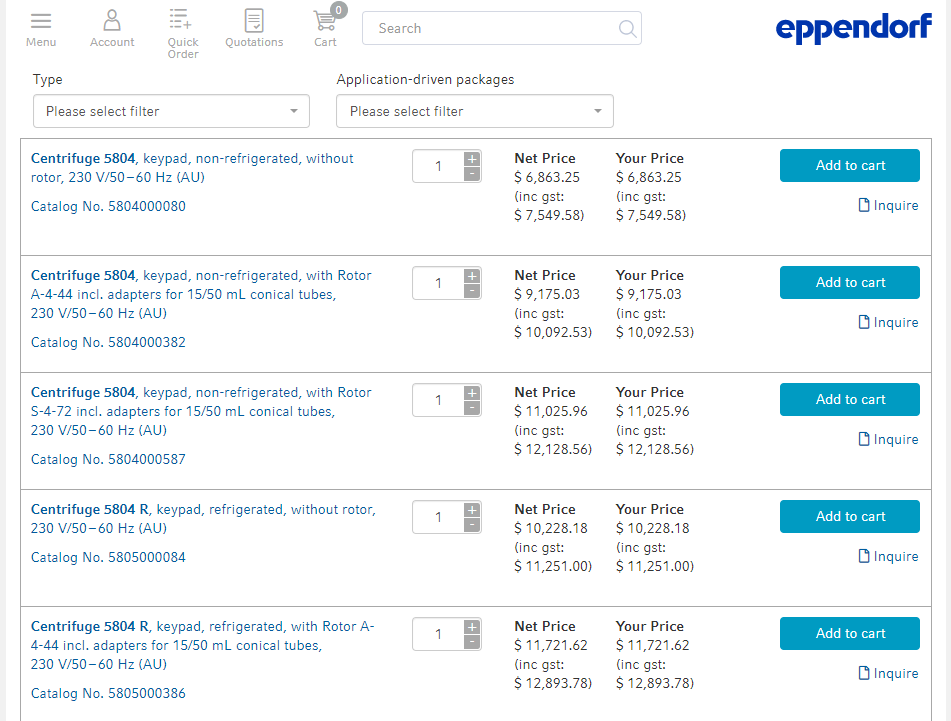


**EDTA**

[EDTA | Sigma-Aldrich (sigmaaldrich.com)](https://www.sigmaaldrich.com/AU/en/search/edta?focus=products&page=1&perPage=30&sort=relevance&term=EDTA&type=product_name)


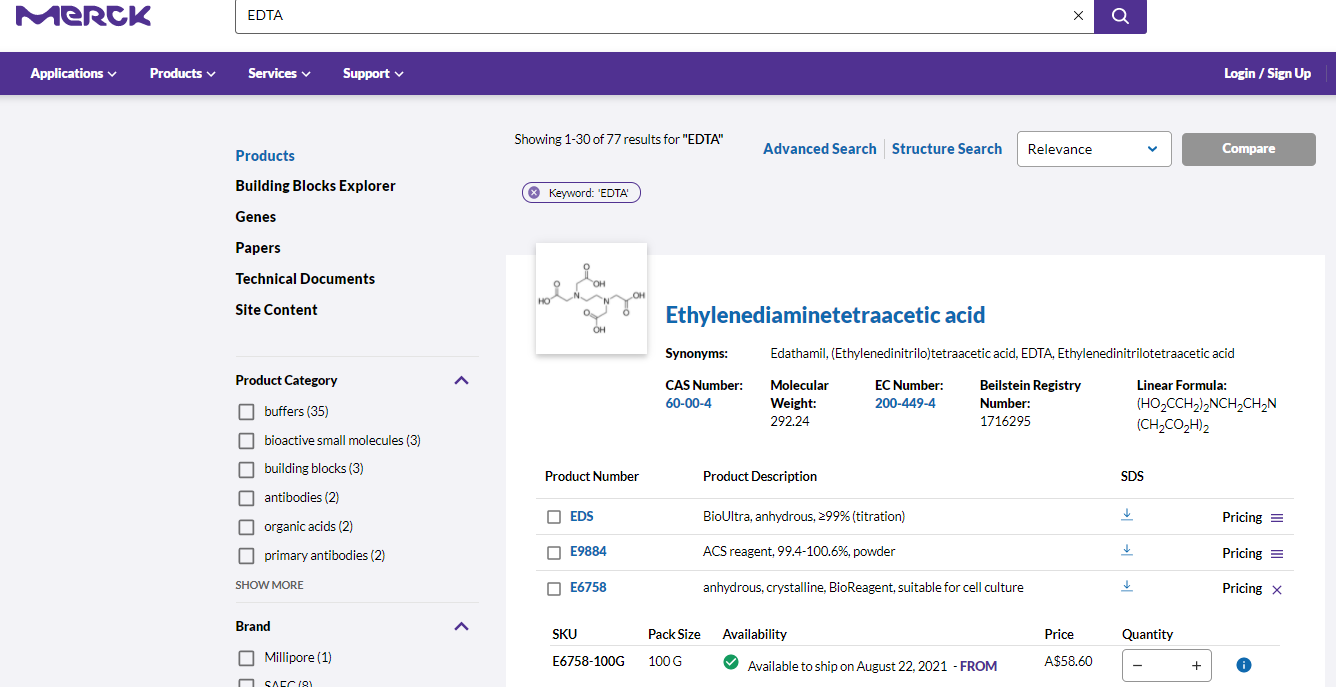


**Styrofoam coolers**

[Polystyrene Six Pack Esky - Rope Handle for Carrying - Foam Sales](https://www.foamsales.com.au/collections/polystyrene-eskies/products/six-pack-esky)


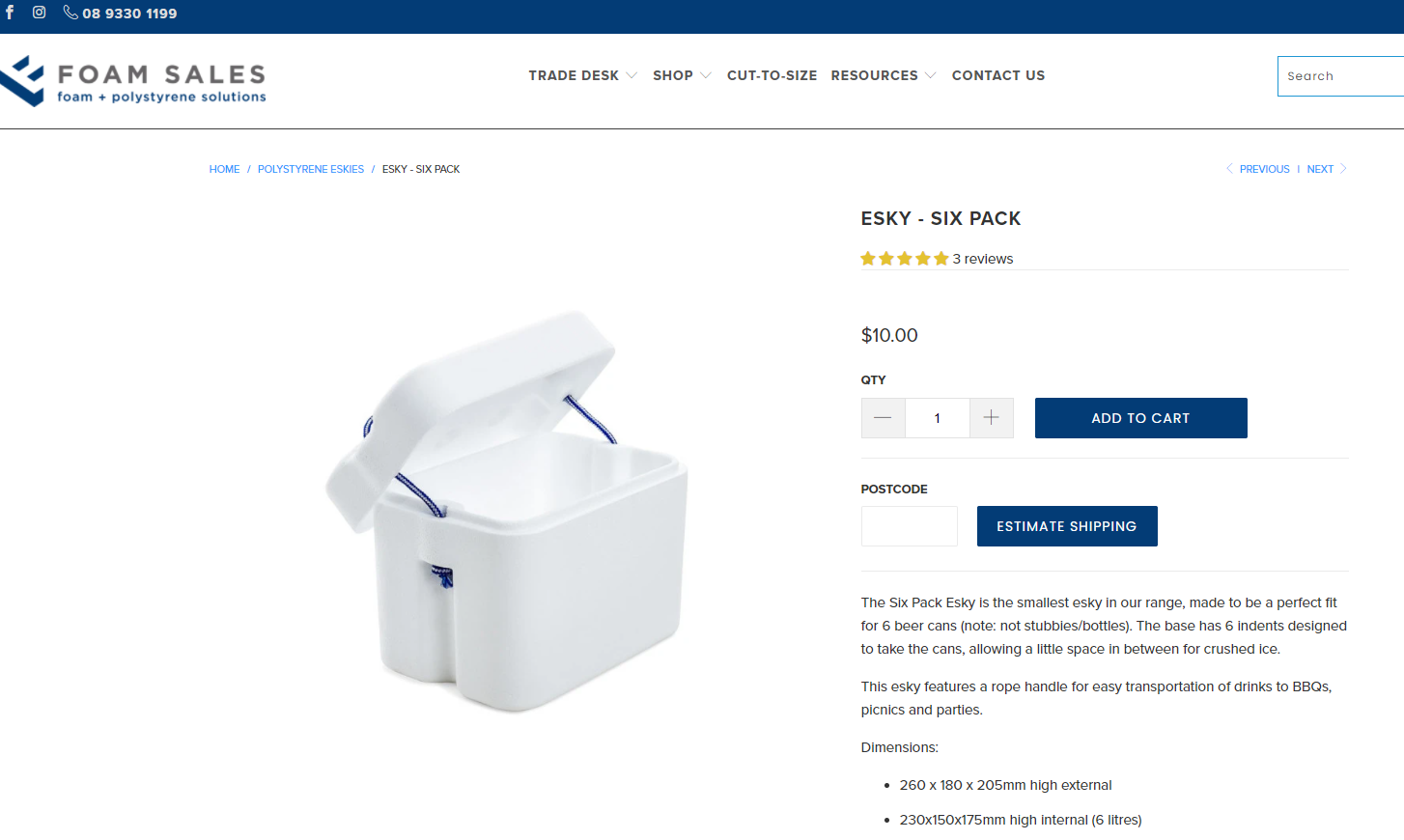


**15ml Centrifuge tubes**

[Conical Centrifuge Tubes, Screw Cap, Graduated, Sterile, 15ml - Buy Online at LabDirect](https://www.labdirect.com.au/conical-centrifuge-tubes-screw-cap-graduated-sterile-15ml-pack-of-500/)


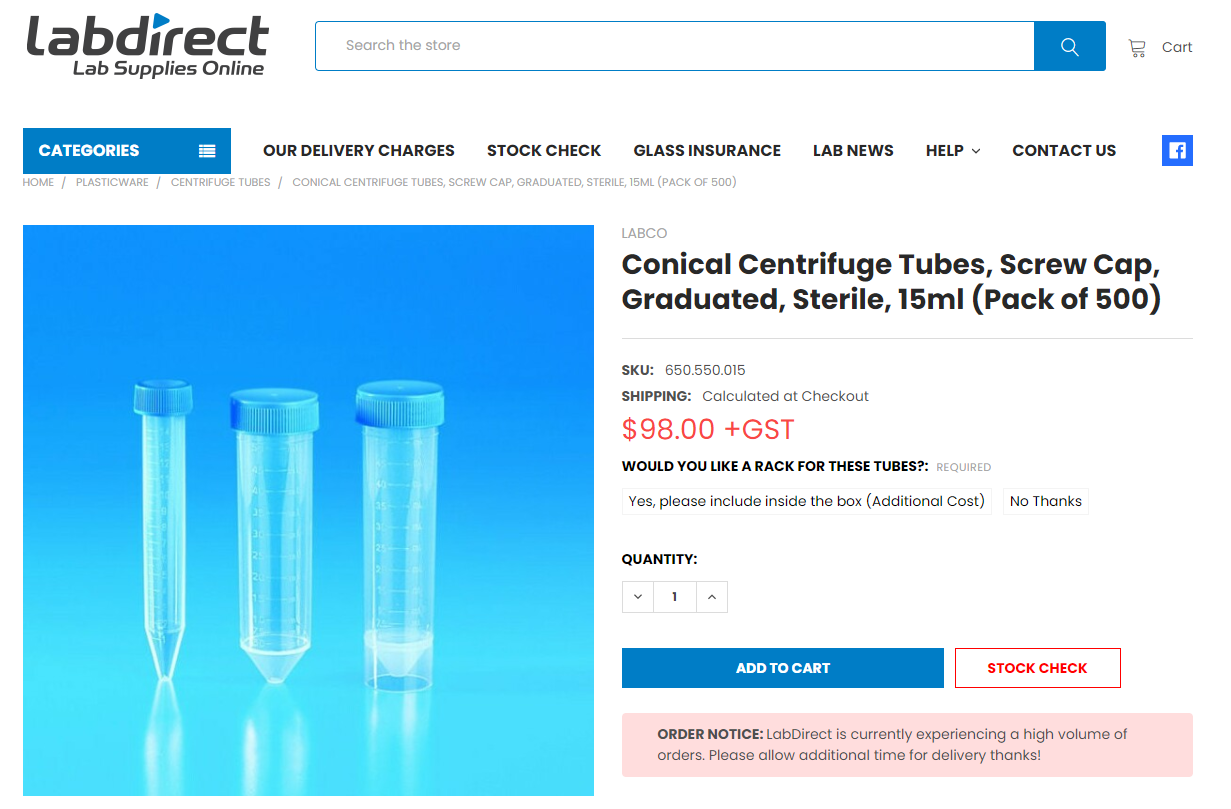


**Formaldehyde solution**

[Formaldehyde solution for molecular biology, 36.5-38% in H2O | 50-00-0 (sigmaaldrich.com)](https://www.sigmaaldrich.com/AU/en/product/sigma/f8775)


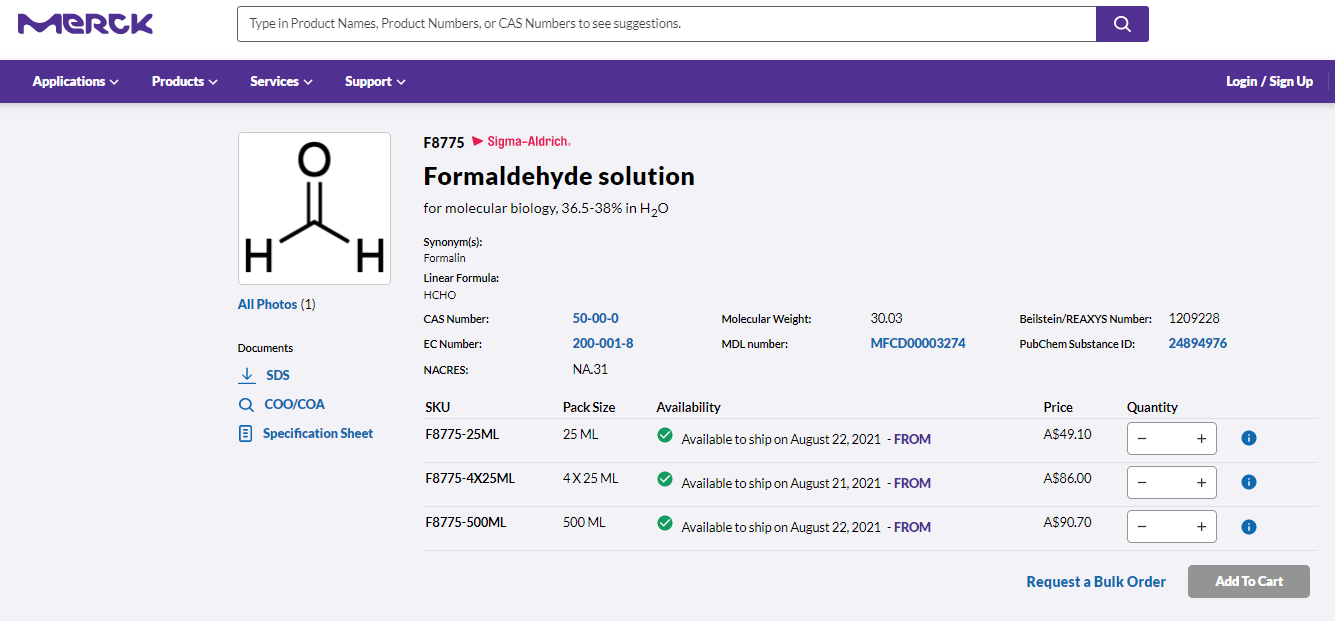


**H2O2 solution**

[Hydrogen peroxide solution 30 % (w/w) in H2O, contains stabilizer | 7722-84-1 (sigmaaldrich.com)](https://www.sigmaaldrich.com/AU/en/product/sigma/h1009)


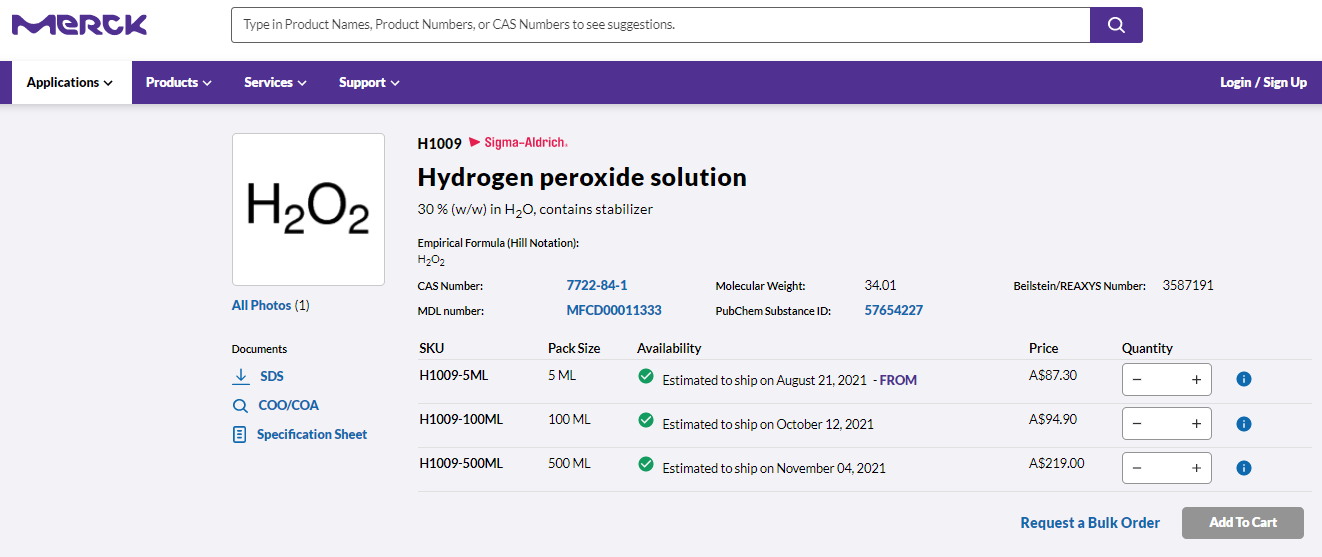


**Homogeniser**

[PRO Scientific Bio-Gen PRO200 Homogenizer](https://proscientific.com/hand-held-homogenizers/bio-gen-pro200-homogenizer/)


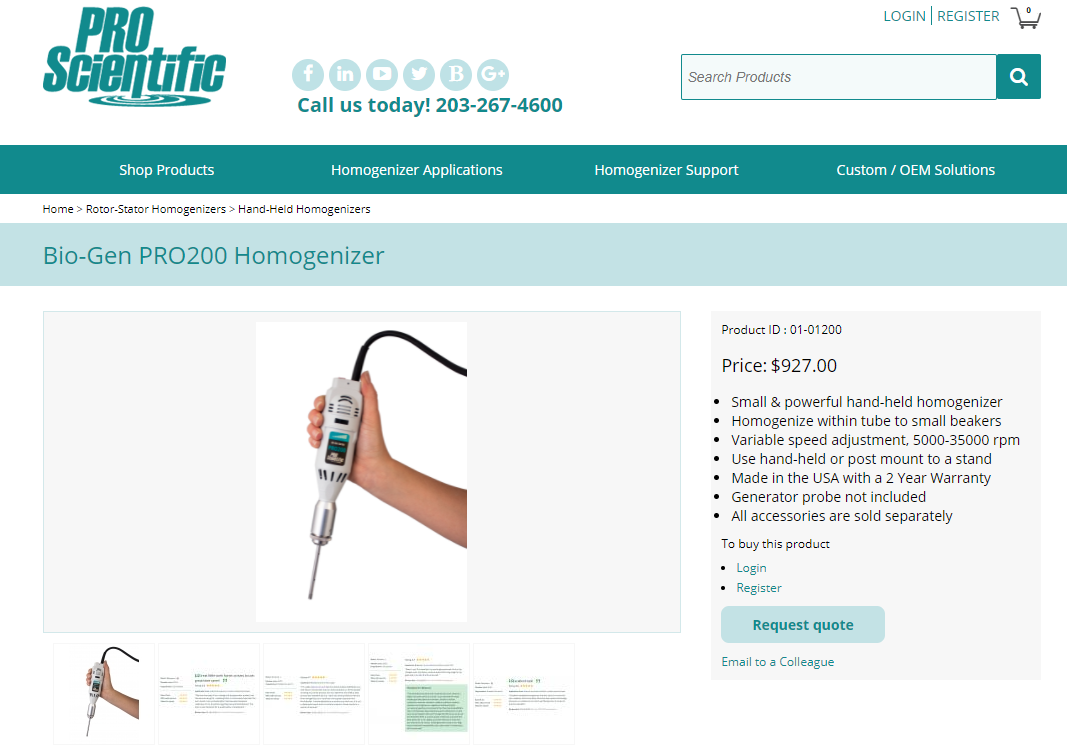


**KimWipes**

[Kimtech® Science™ 34120A KimWipes™ Delicate Task Wipers - <br> White - 280 Sheets/Box - Case of 30 Boxes | KIMTECH SCIENCE* Wipers | Cleaning Cloths, Wipers & Sponges | Hygiene & Cleaning | Blackwoods](https://www.blackwoods.com.au/hygiene-cleaning/cleaning-cloths-wipers-sponges/kimtech-science-wipers/wiper-kimwipes-delicate-34120-21x11cm-30/p/01522596?text=kim+wipes)


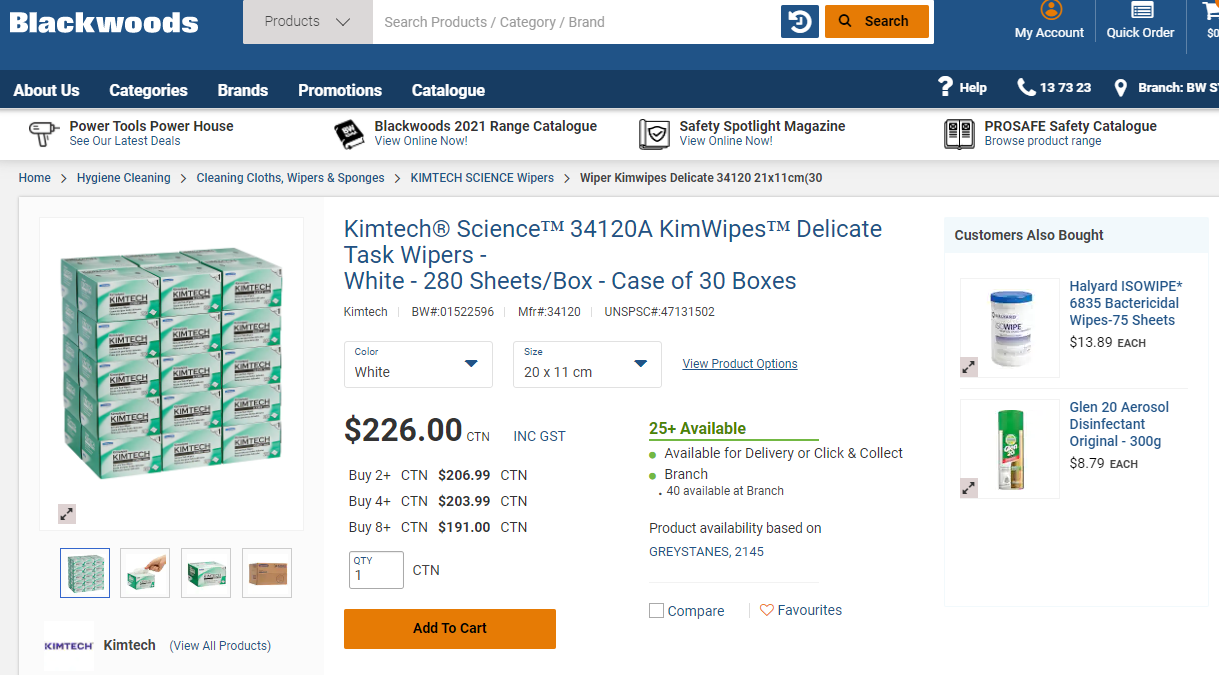


**Zip-lock bags – 4x6”**

[Econo-Zip Reclosable Bags (thomassci.com)](https://www.thomassci.com/Laboratory-Supplies/Bags/_/Econo-Zip-Reclosable-Bags?q=Zip%20Lock%20Bags)


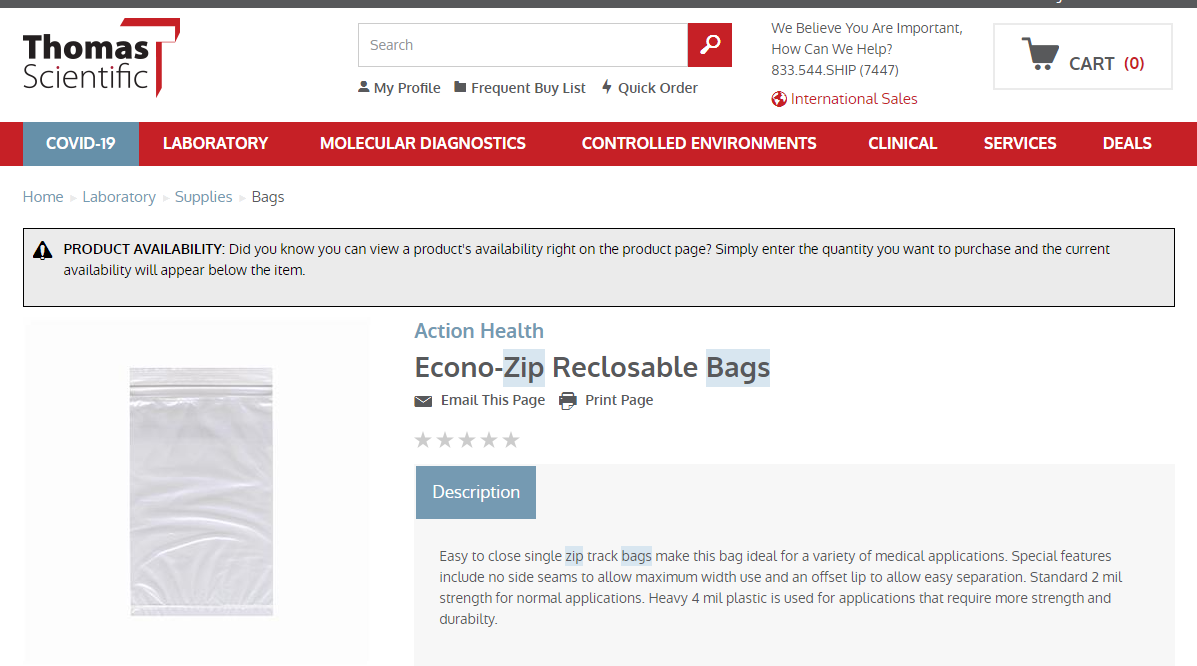


**Microcentrifuge tubes, 1.5ml**

[1.5ml MICROCENTRIFUGE TUBE | Interpath](https://www.interpath.com.au/product/greiner-bio-one/microcentrifuge-tubes/616201_836)


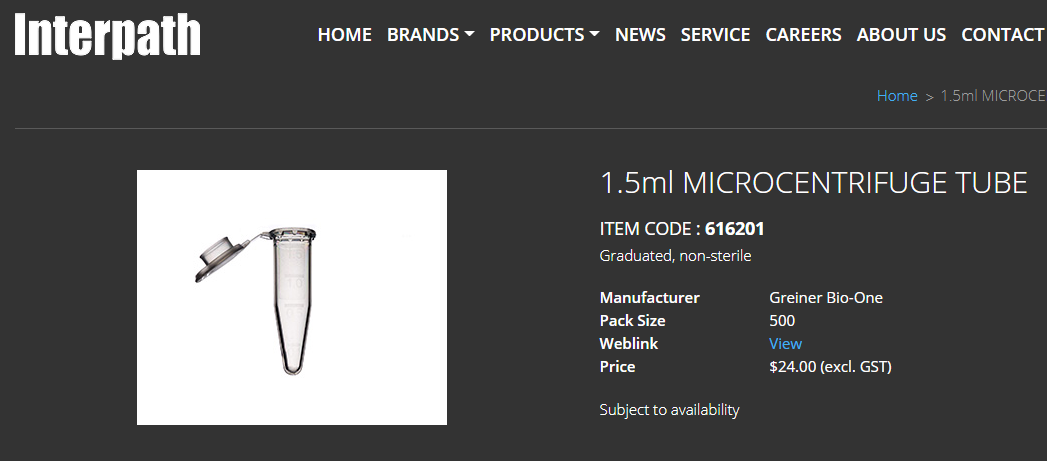


**Multichannel pipettes**

**20uL**


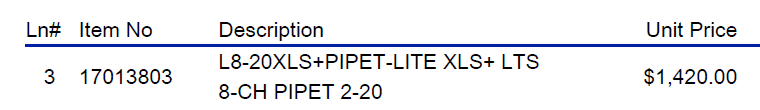


**200uL**


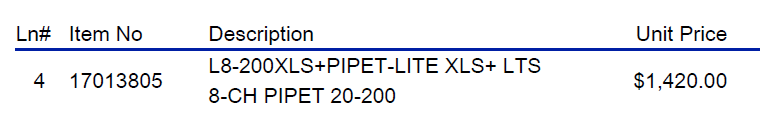


**Sodium Hydroxide pellets**

[Sodium hydroxide - ‘Caustic soda’, Sodium hydroxide solution (sigmaaldrich.com)](https://www.sigmaaldrich.com/AU/en/substance/sodiumhydroxide40001310732)


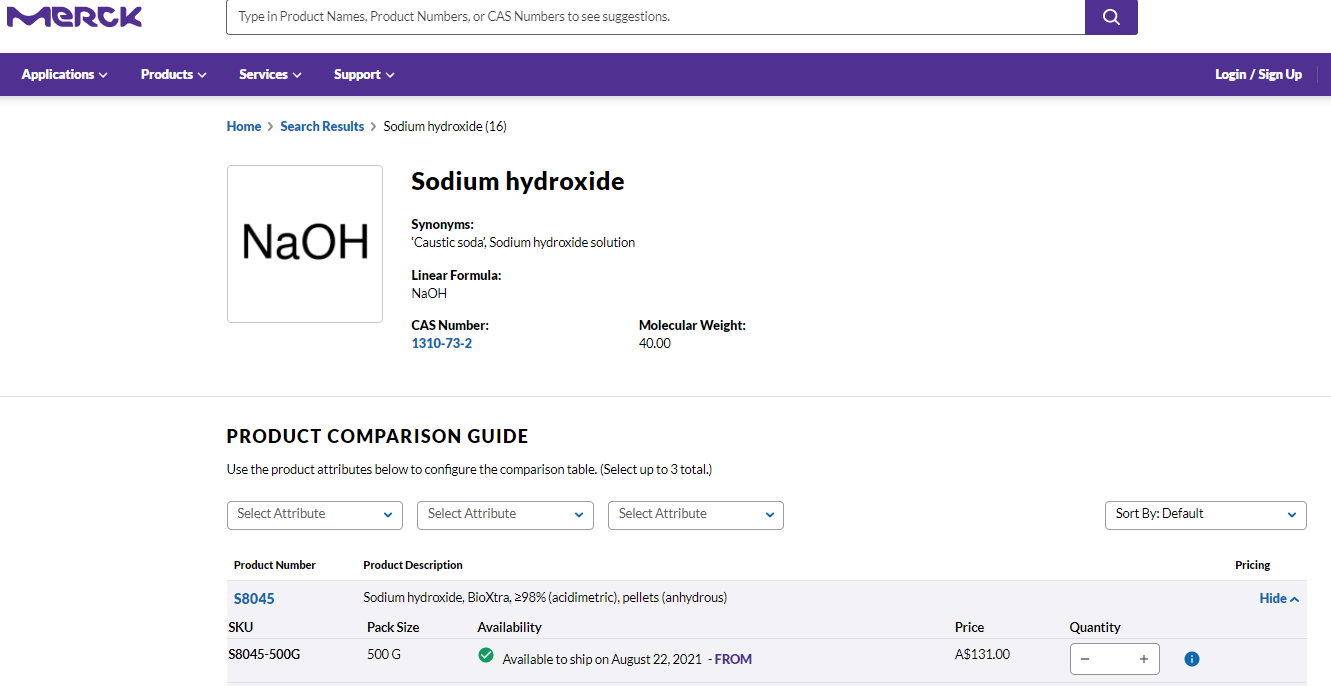


**Oven**


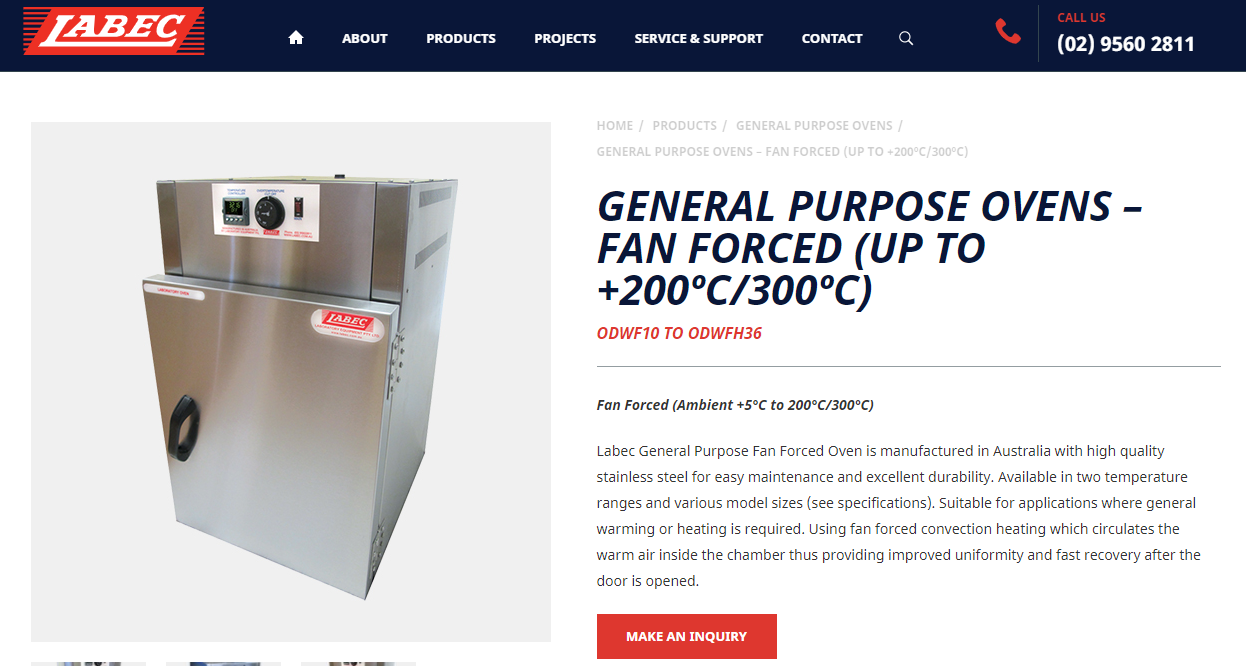


**PBS tablets**

[Phosphate buffered saline - PBS, Phosphate buffered saline (sigmaaldrich.com)](https://www.sigmaaldrich.com/AU/en/substance/phosphatebufferedsaline1234598765)


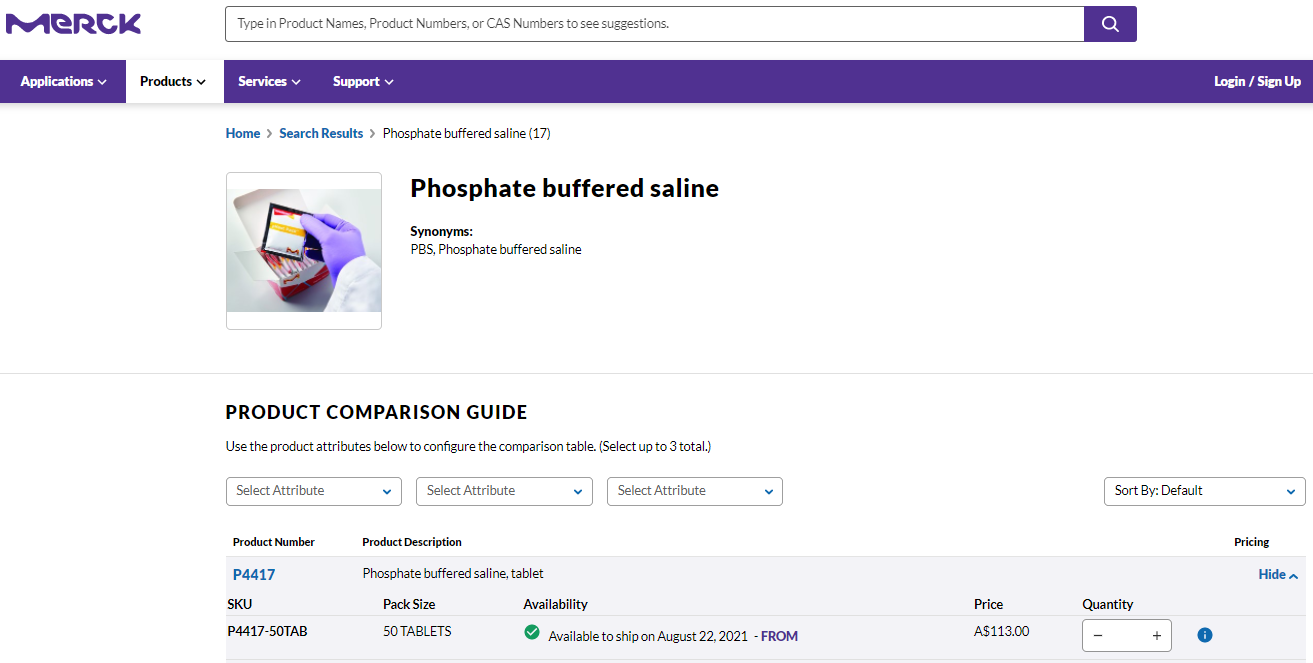


**Single-channel pipettes**


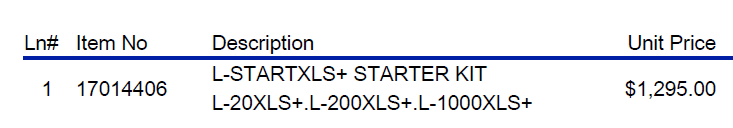


**Protein kit – BD BioRad**


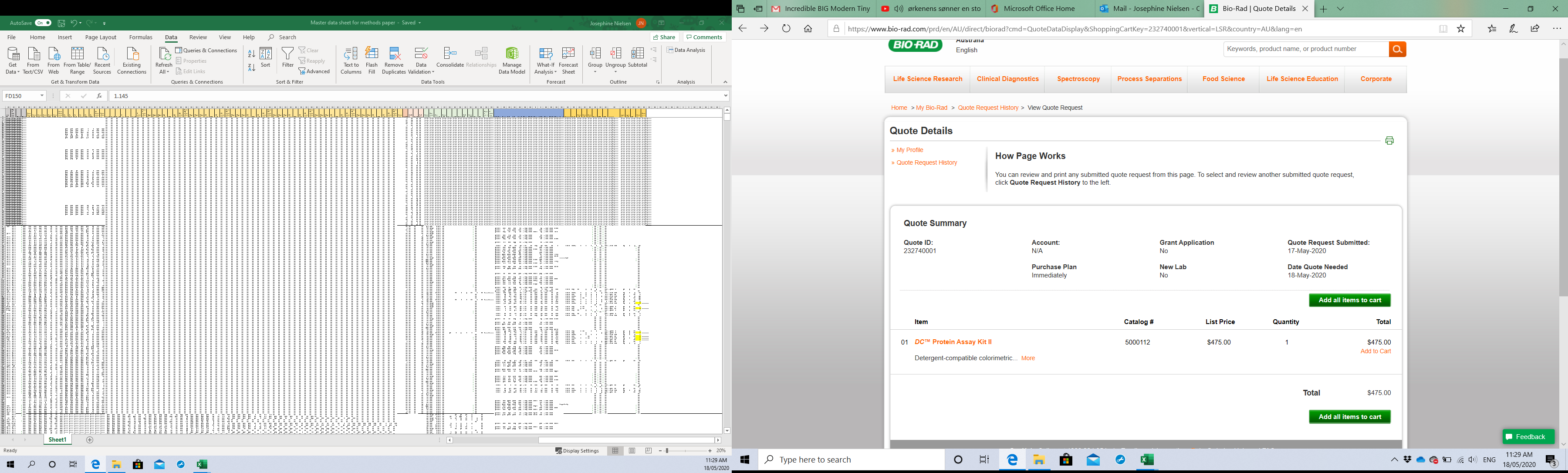


**Small zip-lock bags**

[Small Resealable Bags 90 x 60mm | Seal Bags Small | QIS Packaging](https://www.qispackaging.com.au/bag/resealable-ziplock-bags/resealable-press-seal-bags-50um-and-under/resealable-plastic-bag-90mm-x-60mm)


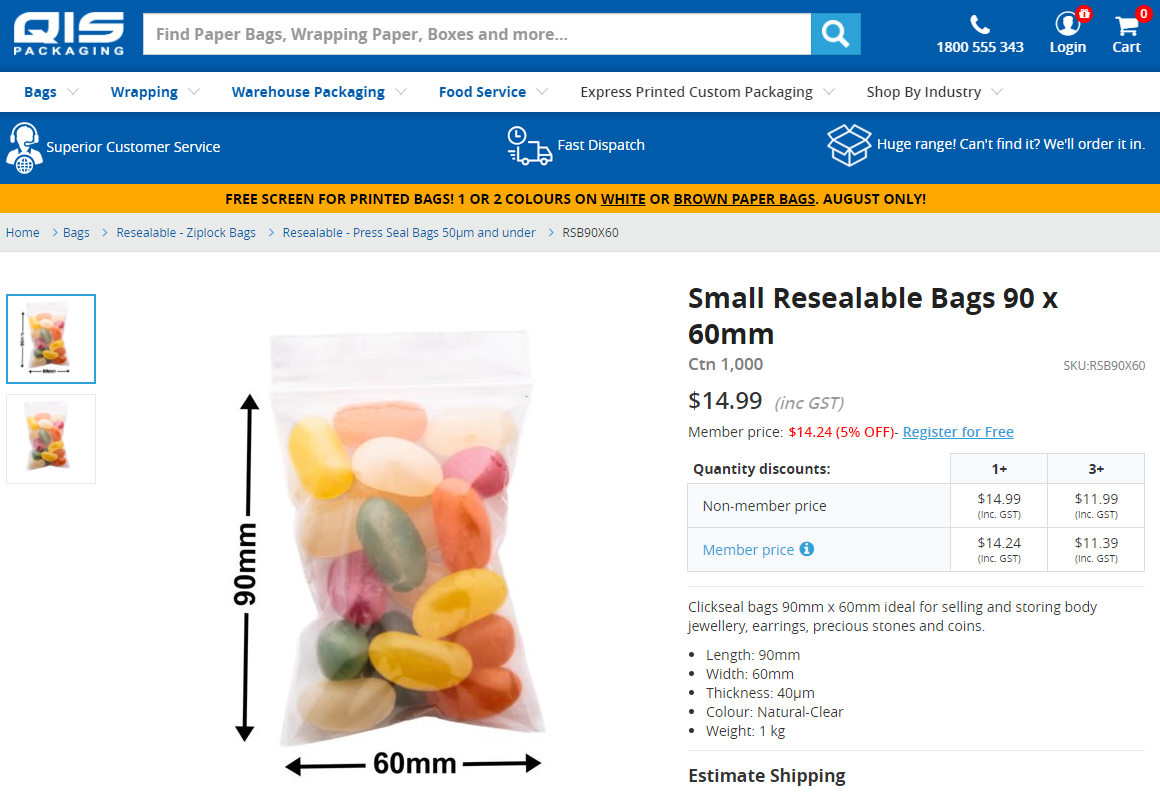


**Digital timer**

[Wiltshire Digital Timer | BIG W](https://www.bigw.com.au/product/wiltshire-digital-timer/p/510475/)


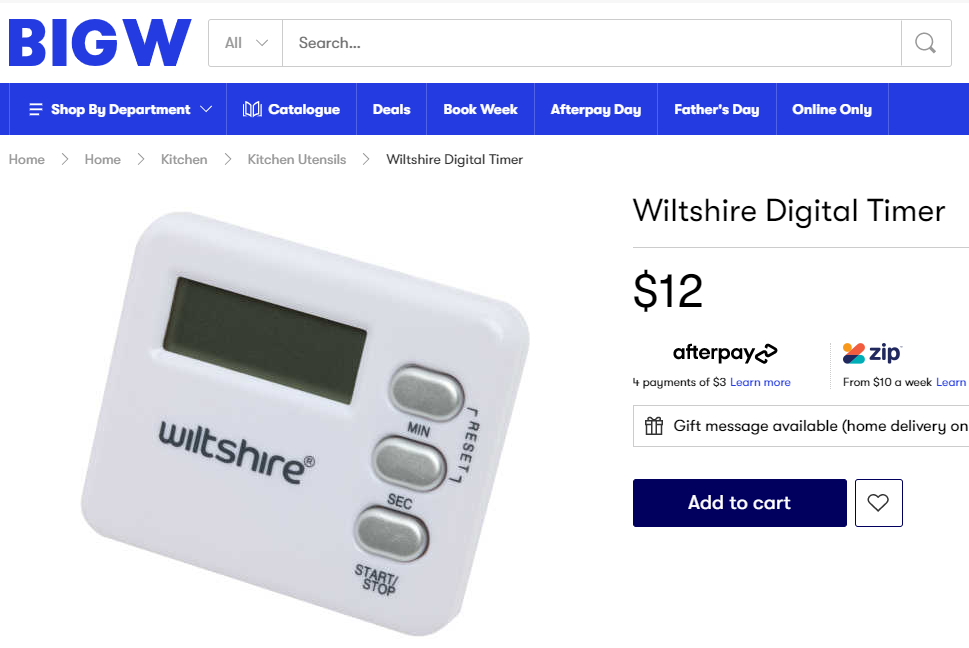


**Vortex mixer**

[Personal Vortex Mixer - VM1 (instrumentchoice.com.au)](https://www.instrumentchoice.com.au/personal-vortex-mixer-scvm1?campaign=355619813&content=&keyword=&msclkid=d680e363ed481fbf7f1382427930a88f&utm_source=bing&utm_medium=cpc&utm_campaign=Shopping%20%7C%20Medium%20%7C%20New&utm_term=4579603370961330&utm_content=Medium)


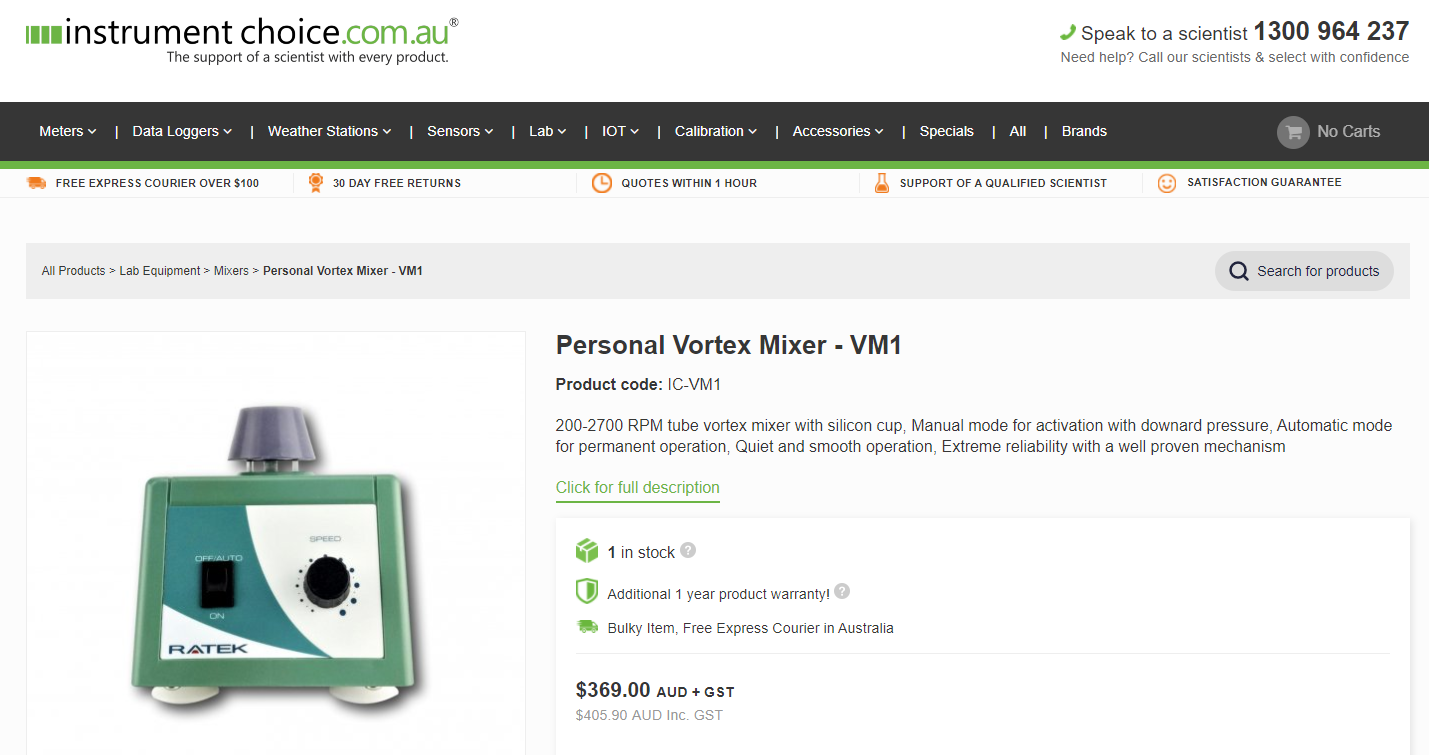


**Multi-channel reservoirs**

[Reagent Reservoirs (thomassci.com)](https://www.thomassci.com/Molecular-Diagnostics/Liquid-Handling/Reservoirs/_/REAGENT-RESERVOIRS1?q=Multi-channel%20Reagent%20Reservoirs)


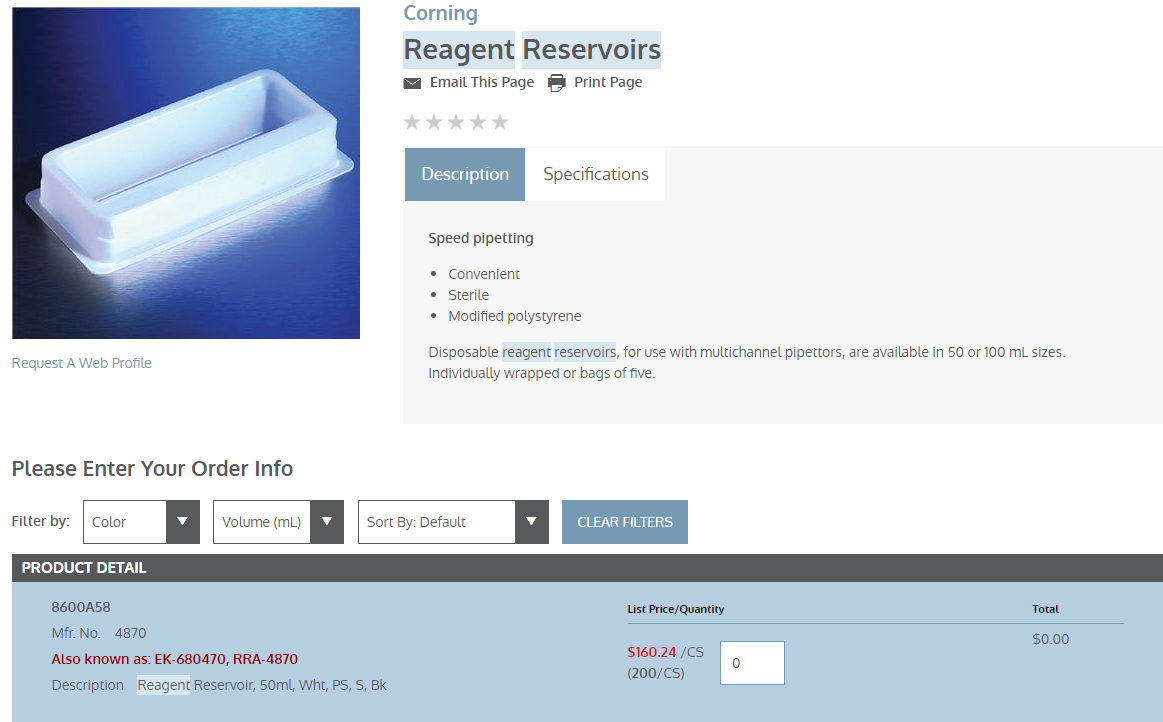


**BD CS&T beads**

[CS&T Research Beads (bdbiosciences.com)](https://www.bdbiosciences.com/en-au/products/reagents/flow-cytometry-reagents/clinical-discovery-research/controls-and-supporting-reagents-ruo-gmp/cs-t-research-beads.650621)


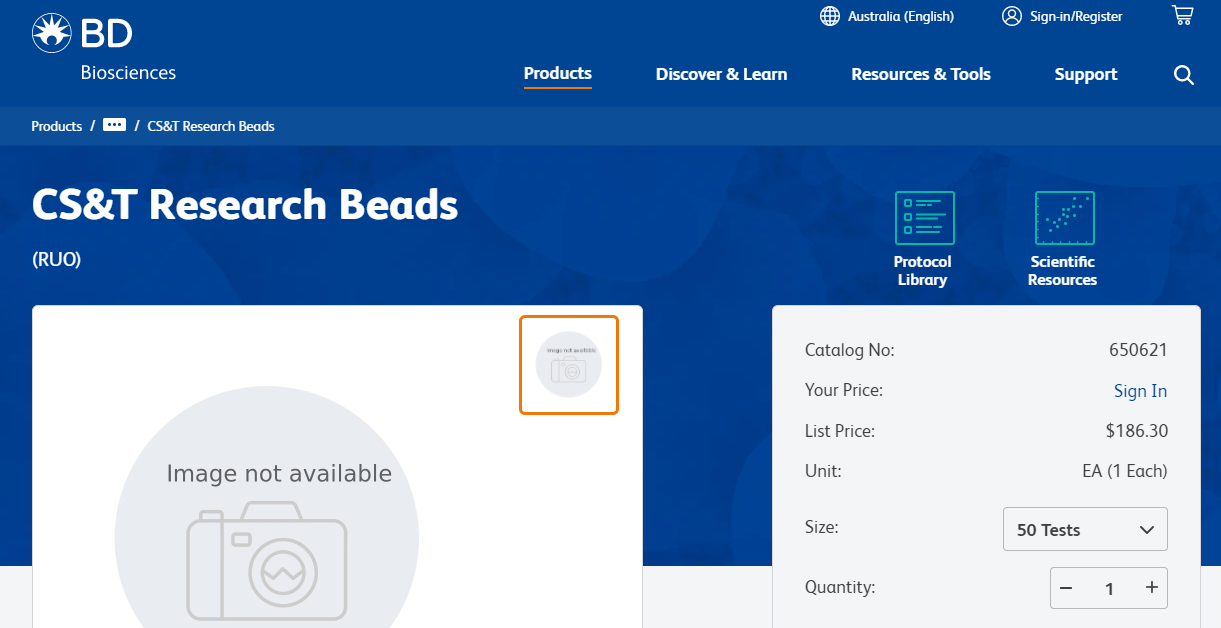


**Colour chart**

[Coral Health Chart – CoralWatch](https://coralwatch.org/index.php/product/coral-health-chart/)


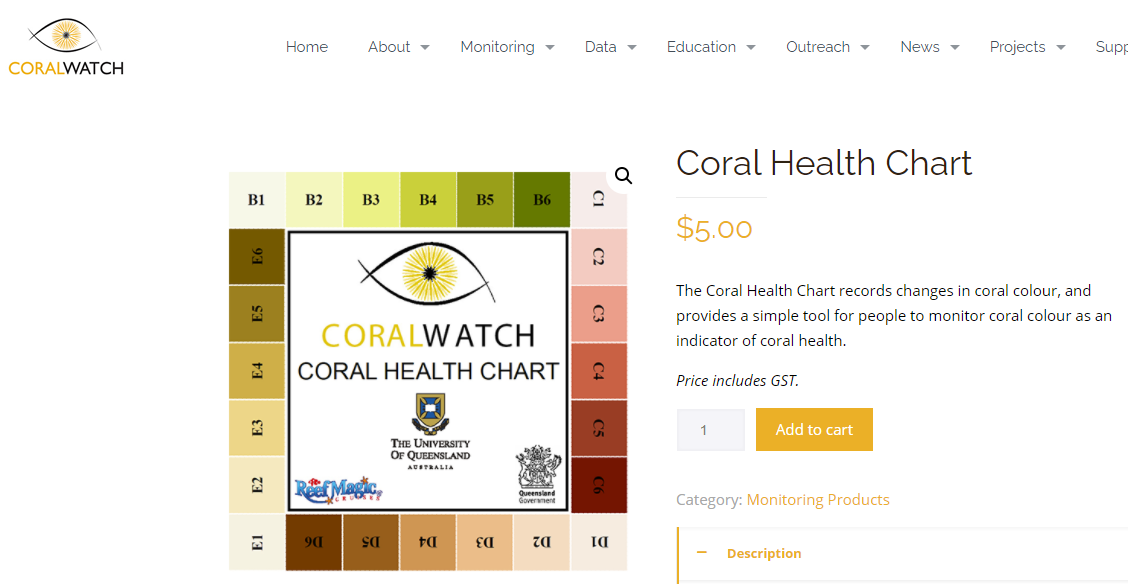


**SD card**

[SanDisk Extreme SDXC Memory Card 128GB Black | Officeworks](https://www.officeworks.com.au/shop/officeworks/p/sandisk-extreme-sdxc-memory-card-128gb-black-sdsdxv5128)


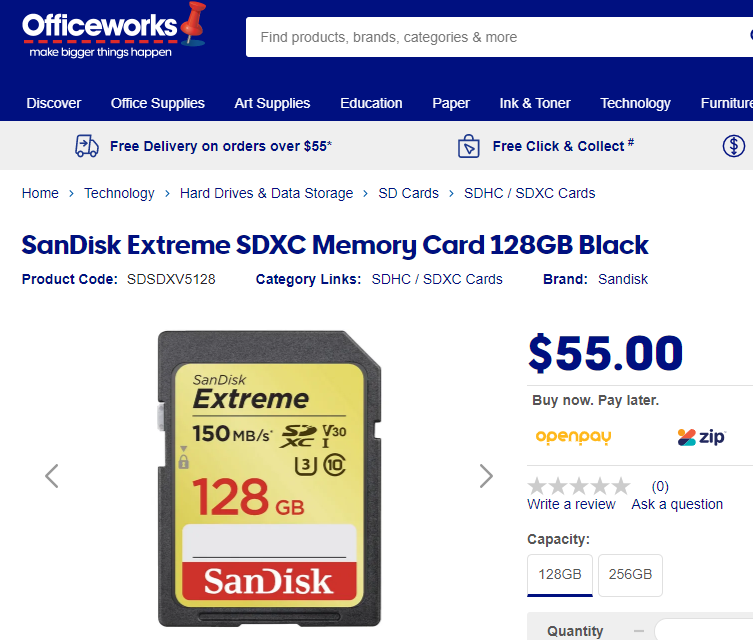


**Camera**

[Discontinued - DSLR D300 - Nikon Australia Pty Ltd](https://www.nikon.com.au/en_AU/product/discontinued/digital-slr-cameras/d300)


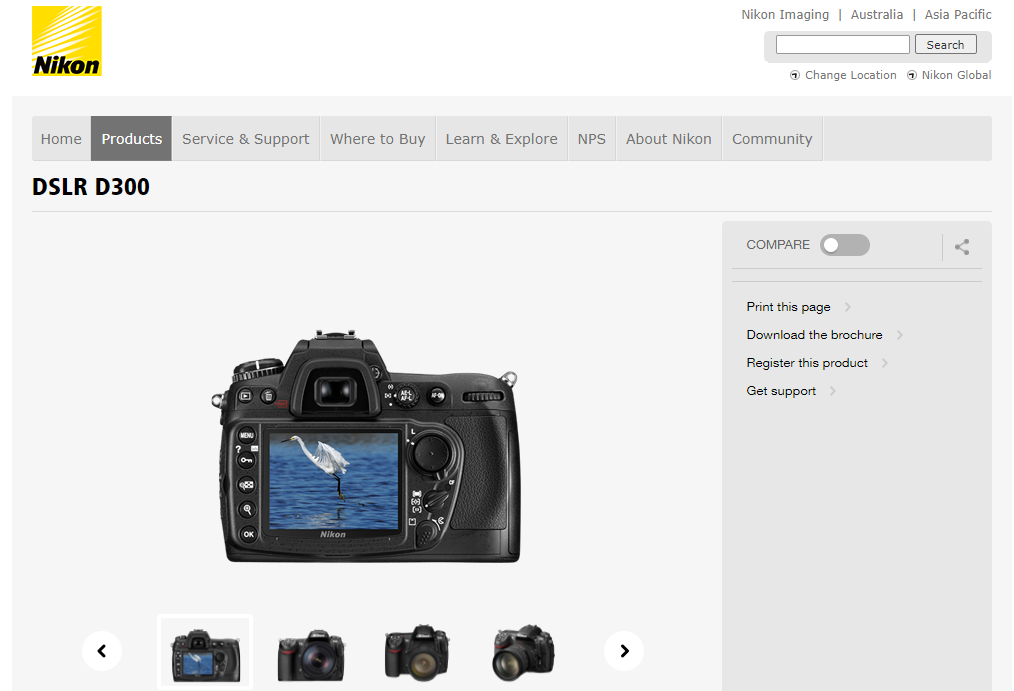


**Scales**

[A&D FX-i Best Ammunition Reloading Scales - Shop Online (scaleshop.com.au)](https://www.scaleshop.com.au/a-d-fx-i/)


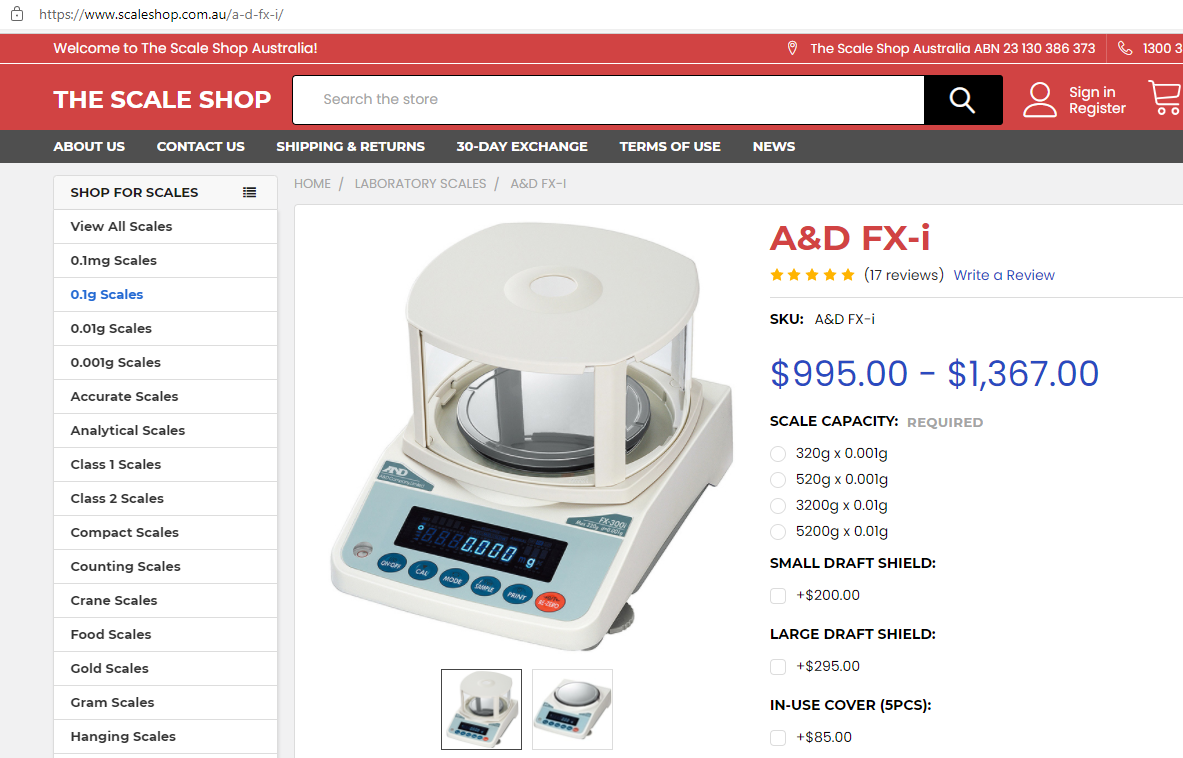


**Forceps**

[Aaxis SM Forceps Stainless Steel Splinter 12.5cm — Medshop Australia](https://www.medshop.com.au/products/aaxis-sm-forceps-stainless-steel-splinter-12-5cm)


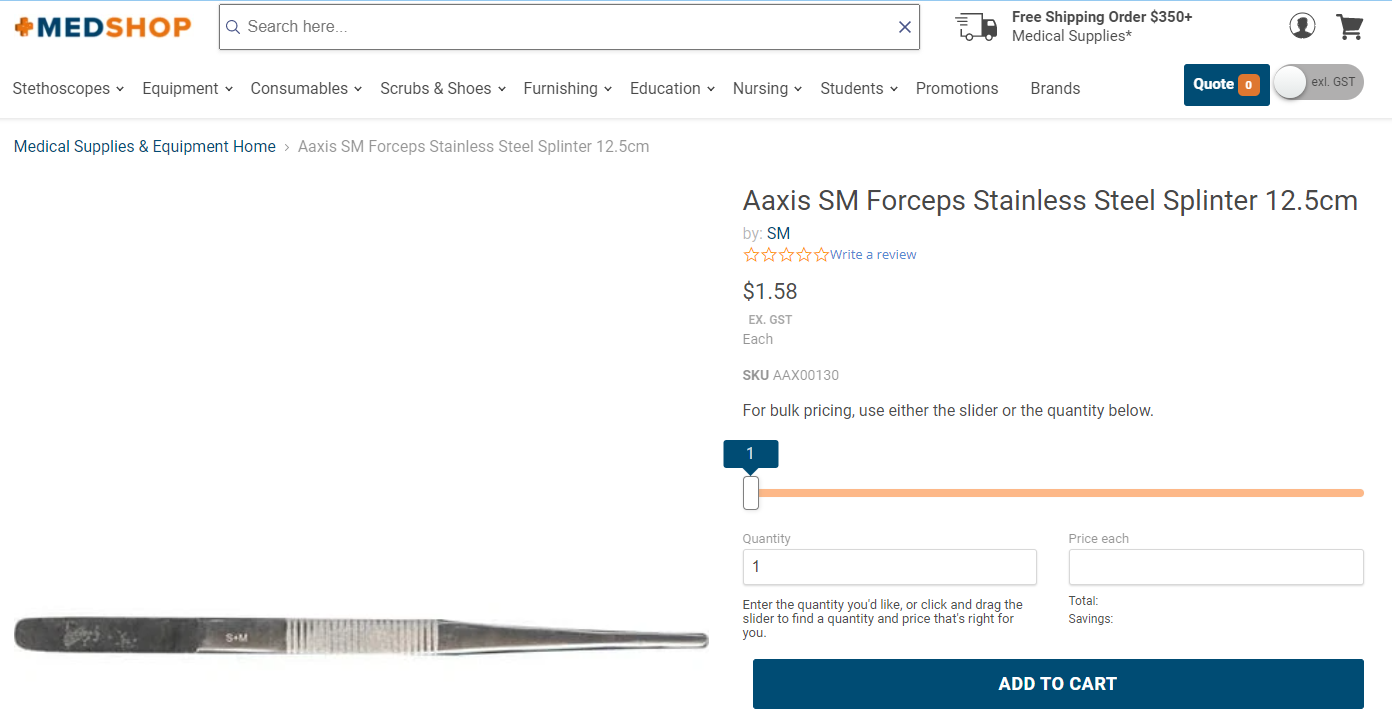


**Paraffin wax**

[We R Memory Keepers 1.3 Kg Wick Paraffin Wax (spotlightstores.com)](https://www.spotlightstores.com/craft-hobbies/other-crafts/candle-making/we-r-memory-keepers-13-kg-wick-paraffin-wax/BP80465106)


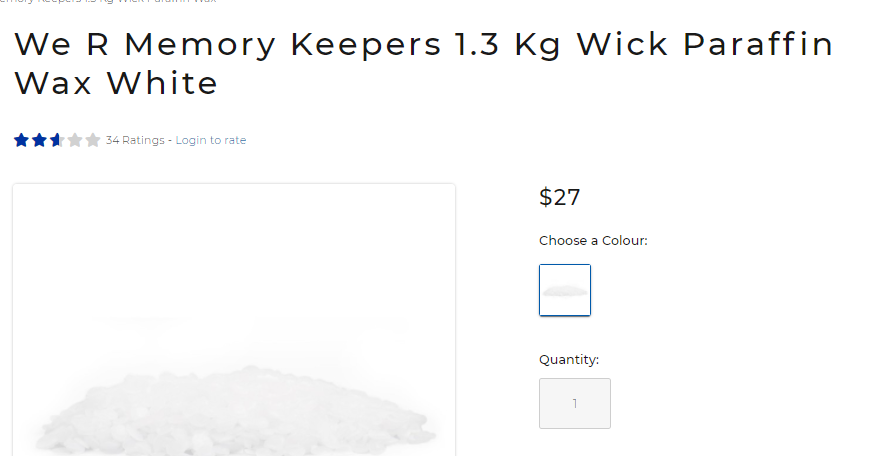


**Waterbath**

[Digital Thermostatic Water Bath 4L (westlab.com.au)](https://www.westlab.com.au/digital-thermostatic-water-bath-4l)


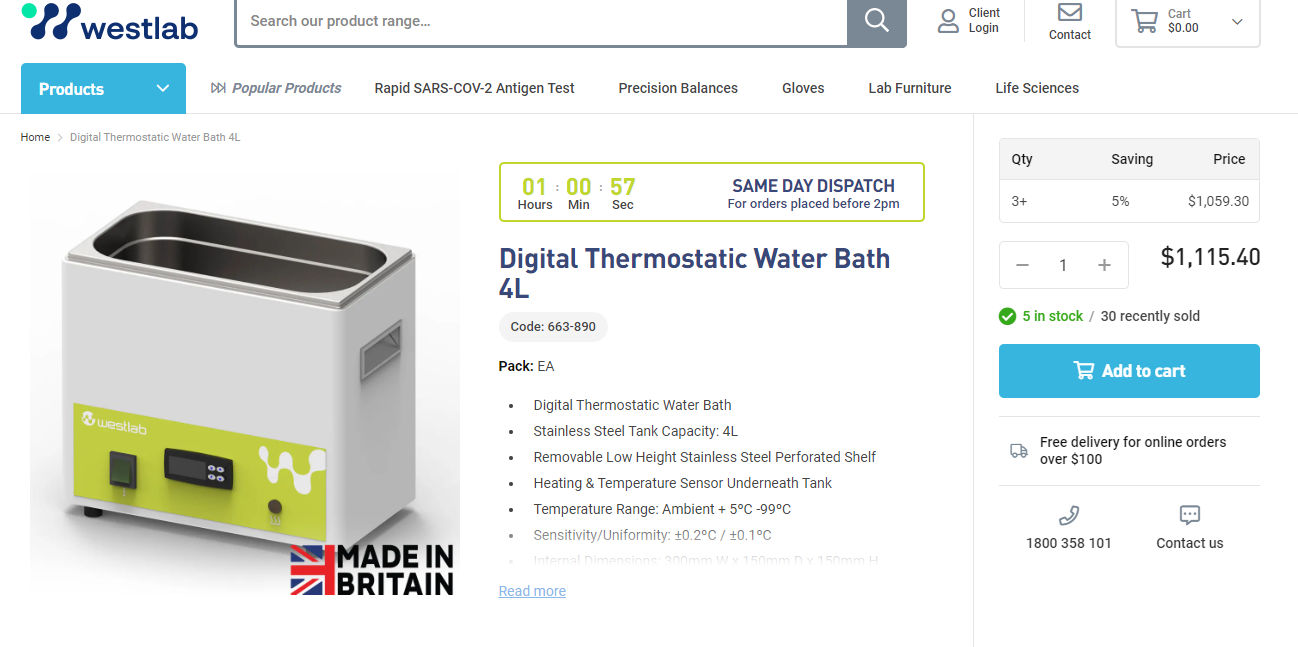

Supplement: Supplementary file 2 — Supplementary Information 2. [file 41598_2022_20138_MOESM2_ESM.docx]
